# *Pseudomonas aeruginosa*-derived volatile organic compounds modulate host immunity to disrupt airway mucus homeostasis

**DOI:** 10.3389/fimmu.2026.1823251

**Published:** 2026-08-26

**Authors:** Shanny Hsuan Kuo, Jaishree Sharma, Cong Wu, Miranda D. Vieson, Beata Kosmider, Scott H. Randell, Som G. Nanjappa, Gee W. Lau

**Affiliations:** 1 Department of Pathobiology, College of Veterinary Medicine, University of Illinois Urbana-Champaign, Urbana, IL, United States; 2 Department of Comparative Medicine, School of Medicine, University of Washington, Seattle, WA, United States; 3 Fred Hutchinson Cancer Center, Seattle, WA, United States; 4 Veterinary Diagnostic Laboratory, College of Veterinary Medicine, University of Illinois Urbana-Champaign, Urbana, IL, United States; 5 Center for Inflammation and Lung Research, Department of Microbiology, Immunology and Inflammation, Lewis Katz School of Medicine, Temple University, Philadelphia, PA, United States; 6 Marsico Lung Institute/Cystic Fibrosis Center, School of Medicine, University of North Carolina at Chapel Hill, Chapel Hill, NC, United States

**Keywords:** goblet cell metaplasia, IL-17A, M1 macrophages, mucus hypersecretion, neutrophils, *Pseudomonas aeruginosa*, volatile organic compounds

## Abstract

Chronic pulmonary diseases, including cystic fibrosis, chronic obstructive pulmonary disease, and chronic bronchitis, as well as ventilator-associated pneumonia, are characterized by persistent infection of mucus-laden airways. *Pseudomonas aeruginosa* (PA) is a dominant pathogen in these conditions and produces volatile organic compounds (VOCs) that have been proposed as biomarkers of disease exacerbation; however, their immunopathogenic roles remain unclear. We investigated PA-derived VOCs using human bronchial epithelial air–liquid interface cultures and murine models. VOCs exposure significantly increased airway mucin expression and induced a proinflammatory response characterized by M1 macrophage polarization (iNOS^+^), neutrophil recruitment, and expansion of IL-17A–producing Thy1.2^+^ lymphocytes. Functional depletion of macrophages, neutrophils, or IL-17A *in vivo* each attenuated mucin production and goblet cell metaplasia, indicating non-redundant contributions to mucus pathology. *In vitro*, IL-17A neutralization partially restored FOXA2 expression and reduced mucin production, supporting a role in mucus regulation. On the mechanistic level, we discovered an IL-17A-dependent dual-axis pathway involving both epithelial cell-mediated autocrine and lymphocyte-mediated paracrine signaling that contributes to the feed-forward loop of inflammation and enhances the signaling pathways regulating mucus hypersecretion in airways. We conclude that PA VOCs activate multiple proinflammatory responses to convergently drive mucus pathogenesis in the diseased lung.

## Introduction

Pulmonary diseases, including cystic fibrosis (CF), chronic obstructive pulmonary disease (COPD), and ventilator-associated pneumonia (VAP), are marked by persistent airway infections, goblet cell metaplasia and hyperplasia, and muco-obstruction (1, 2). *Pseudomonas aeruginosa* (PA) is an important pathogen of these diseased airways. PA is metabolically versatile and produces various secondary metabolites, which may modulate the respiratory epithelium physiology and mucosal immunity (3, 4). Extensive studies using breath, sputum, and bronchoalveolar lavage fluid (BALF) samples from patients with cystic fibrosis (CF) have identified several major volatile organic compounds (VOCs) produced by PA, including hydrogen cyanide (HCN) (5, 6), 2-aminoacetophenone (2-AA) (7), 2-nonanone (7–10), 1-undecene (11), and methyl thiocyanate (12). In addition, 1-dodecene and 1-undecene have been detected in the breath of smokers with a predisposition to chronic obstructive pulmonary disease (COPD) (9). Beyond their value as noninvasive diagnostic biomarkers, increasing evidence indicates that PA-derived VOCs exert biologically active effects on host cells. For instance, 2-AA modulates bacterial virulence gene expression through the multiple virulence factor regulator (MvfR) system, thereby suppressing acute virulence while promoting chronic infection traits (13), and has also been shown to impair ATP production in host skeletal muscle (14, 15). Similarly, 2-undecanone induces neutrophil apoptosis and inhibits innate immune responses (16), including LPS-induced activation, thereby weakening host antimicrobial defense. However, although there is emerging knowledge on the immunopathological functions of PA-derived VOCs, it remains unclear how they influence airway mucus biology.

Mucus hypersecretion and goblet cell metaplasia (GCM) are common responses to airway inflammation caused by allergens and/or microbial infection in asthma, CF, COPD, VAP, and other inflammatory respiratory diseases (2). Several pathways promote GCM by modifying the developmental fate of airway basal cells. For example, interleukin (IL)-17, produced mainly by T helper (Th)17 cells, causes neutrophilic airway inflammation to exacerbate the severity of asthma and COPD by promoting GCM via a NOTCH2-dependent signaling in airway basal cells (17). In allergen-induced asthma attack, the IL-4/IL-13-STAT6-SPDEF pathway activates both goblet cell differentiation and Th2 inflammatory responses (18–20), which redirects the fate of airway basal cells-derived progenitor club and ciliated cells towards goblet cell lineage by inducing the NOTCH signaling (17, 21). Other Th2 cytokines, IL-5 and IL-9, also promote GCM and mucin expression (22). Also, EGFR (23–25) and p38 MAP kinase (26) induce GCM and transcription of mucin genes. Importantly, activated STAT6 and EGFR convergently inhibit the expression of FOXA2, a key transcriptional regulator of airway mucus homeostasis (24, 27–32). FOXA2 maintains healthy levels of airway mucus by inhibiting SPDEF and Th2 cytokine-mediated lung inflammation (19, 27, 33). Mouse airway-specific deletion of the *FOXA2* gene causes GCM, excessive mucus, emphysema, and neutrophilic infiltration (27). Further, the concentration of Th2 cytokines is elevated within CF airways (34), which may activate STAT6 to inhibit FOXA2. Notably, we and others have shown that FOXA2 expression is reduced in airways of human and canine patients with COPD (30, 35), bronchopulmonary dysplasia (27), bronchiectasis (27), and asthma (33). Also, cigarette smoke, a major cause of COPD, suppresses FOXA2 expression in a bronchial epithelial cell line and in mice (36). Finally, we have shown that another PA secondary metabolite, pyocyanin, suppresses FOXA2 by activating the antagonistic STAT6 and EGFR signaling pathways, leading to goblet cell hyperplasia, increased MUC5AC and MUC5B mucin expression, and mucus hypersecretion in airway epithelial cells (4, 24, 28–30, 35, 37). Together, these findings highlight FOXA2 as a central integrator of inflammatory and epithelial signaling in mucus homeostasis. Based on these observations, we hypothesized that PA-derived VOC metabolites —specifically 2-AA, 1-undecene, 2-nonanone, and 1-dodecene—modulate immune cell populations and epithelial signaling pathways to suppress FOXA2 activity, thereby promoting GCM and mucus hypersecretion in diseased airways.

## Methods

A complete description of experimental methods is provided in the **Supplementary Materials**. Pertinent methods unique to this study are provided below.

### Chemicals, reagents, and antibodies

VOCs, including 2-AA (W390607), 1-Dodecene (D221600), 1-Undecene (242527), and 2-Nonanone (108731), were purchased from Sigma-Aldrich. Compounds were suspended in DMSO and serially diluted to desired concentrations in PBS (final DMSO concentration < 0.1%). Antibodies utilized in this study include: Santa Cruz Biotechnology (FOXA2, sc-101060; MUC5AC, sc-21701; MUC5B, sc-20119; IL-17R, sc-376374; IL-17A, 740021M and PA5-114455; CC10, sc-365992); Invitrogen (acetyl-α tubulin, 6-11B-1); Cell Signaling Technology (FOXA2, D56D6); Bioss Antibodies (MUC5AC, bs-7166R); and Novus Biologicals (MUC5B, NBPI-92151). Antibodies were diluted in 5% bovine serum albumin, used at dilutions recommended by manufacturers, and optimized as needed. IL-17A neutralizing antibody (IL-17A mAb, 12047-M237) was purchased from Sino Biological, Inc.

### Healthy, CF, and COPD human and canine lung tissues

Lung tissue sections from COPD or control organ donors were provided by Dr. Beata Kosmider at Temple University. De-identified CF lung tissue sections were provided by Dr. Scott Randell at the University of North Carolina at Chapel Hill. The diseased lung sections demonstrated confirmed clinical infections with PA, characterized by 3+ mucoid and 3+ smooth forms. Lung specimen from a COPD dog that died of PA infection was previously described (35) and provided by Dr. Miranda Vieson at the University of Illinois at Urbana-Champaign (UIUC) Veterinary Diagnostic Laboratory.

### Mouse study approval

Mouse experiments were conducted in strict compliance with the guidelines outlined in the “Guide for the Care and Use of Laboratory Animals” by the National Institutes of Health. All mouse experiment procedures were approved by the UIUC Institutional Animal Care and Use Committee (IACUC) under protocols 20120 and 23132.

### Mouse models

All experiments were performed with 6–8 week-old age and sex-matched male and female C57BL/6 mice procured from Charles River Laboratories. IL-17A KO mice (in C57BL/6 genetic background) were originally provided to Dr. Nanjappa by Professor Yoichiro Iwakura (Tokyo University of Science, Tokyo, Japan). For the VOCs challenge, isoflurane-anesthetized mice (groups of 8) received intranasal instillation of specified concentrations of VOCs once daily for 1 to 3 weeks, using a mouse model of chronic inflammation (24, 28, 30, 38). Control mice were intranasally-exposed to the same volume of vehicle (0.1% DMSO in PBS) for the same duration. The concentrations of VOCs intranasal instillation were based on the maximal levels detected in the headspace of bacterial culture (9, 42): 2-AA (26 μg/mL), 1-undecene (317.5 ng/mL), 2-nonanone (22.4 ng/mL), and 1-dodecene (9.5 ng/mL). Mouse airways were evaluated for the degrees of GCM and mucin production using PAS, IHC, and/or IF analyses for MUC5AC, MUC5B, FOXA2, IL-17R, and IL-17A.

### Sex as a biological variable

Our study examined male and female animals, and similar findings are reported for both sexes.

### Macrophages and neutrophils depletion

For macrophages depletion, clodronate liposome (CL) or control empty liposome (EL) (F70101C-N and F70101-N, respectively, FormuMax Scientific) was intraperitoneally injected at 10 μL/g mouse weight on Day -4, and subsequent injection of 5 μL/g intraperitoneally on Days 1 and 5. VOCs were administered intranasally once daily. For neutrophils depletion, mice were intraperitoneally injected with rat anti-mouse Ly6G (Clone 1A8, BioXCell) on Days 0, 2, 4, and 6, prior to daily exposure to VOCs. Control mice received rat IgG2a isotype control (Clone 2A3, BioXCell). The mice were sacrificed on Day 8.

### Flow cytometric analysis

Bronchoalveolar lavage fluid (BALF) and lungs were harvested from inoculated mice at 3 weeks post-inoculation. Flow cytometric analyses of single-cell suspensions of BALF and lung homogenates were performed as described (39, 40).

### Airway cell cultures

Submerged and ALI cultures of the human bronchial epithelial 16HBE14o- cells were grown as previously published (30). Airway cells were exposed to VOCs for 24–48 hours and subjected to western blotting or fluorescence microscopy analyses.

### Periodic acid-Schiff, immunofluorescence, and immunohistochemistry staining

Various staining, image acquisition, and quantification of mouse, human, and canine lung sections were performed as previously published (29, 30, 38). The degree of PAS-stained mucin expression was quantified using a 4-tier goblet cell score grading system described by Grundström et al. (41),: Grade 0: No goblet cell observed per bronchiole. Grade 1: Up to 5 goblet cells per bronchiole. Grade 2: 5–20 goblet cells per bronchiole; Grade 3: More than 20 goblet cells per bronchiole.

### Whole-cell, cytosolic, and nuclear protein preparations and Western blotting analyses

Protein extraction and western blotting were performed as previously published (24, 30, 31, 35).

### Statistical analysis

Statistical analyses were conducted using GraphPad Prism 9.4.1 and 10. Quantitative data were presented as the mean ± standard error of the mean (s.e.m). In cases where equal variance was observed among samples, the significance between groups was assessed through a two-tailed Student’s t-test. For samples exhibiting unequal variance, a two-tailed Welch’s t-test was employed. In scenarios involving multiple comparisons, a one-way analysis of variance (ANOVA) followed by a *post hoc* Tukey’s test was implemented. Significance levels were established as follows: *p ≤ 0.05, **p ≤ 0.01, ***p ≤ 0.001, and ****p ≤ 0.0001.

## Results

### PA VOCs attenuate FOXA2 expression and induce mucin production

**Figure 1:**
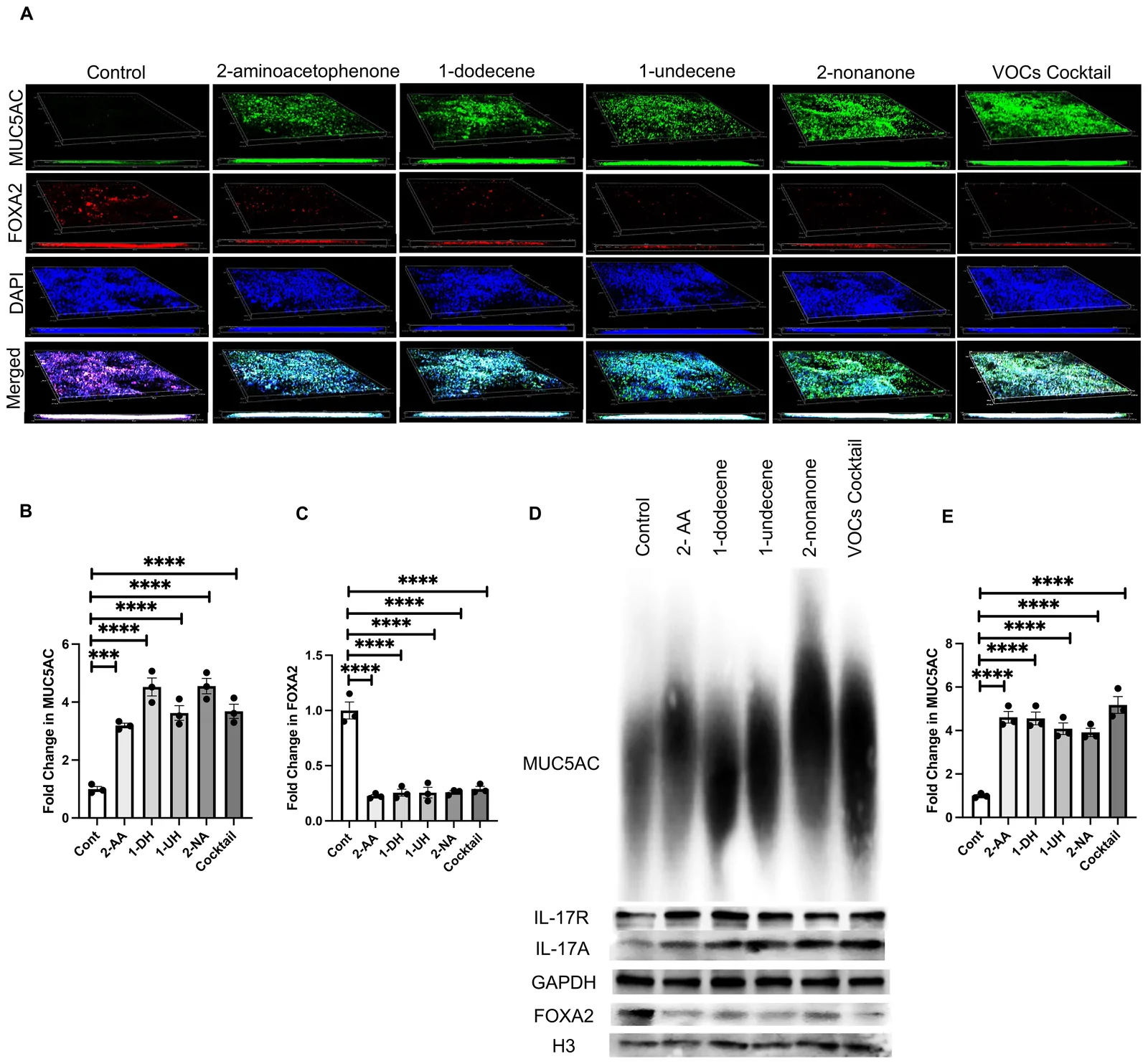
PA-derived VOCs attenuate FOXA2 expression and induce mucin production in submerged and air-liquid interface (ALI) cultures of 16HBE14o- cells. **(A)** Double immunofluorescence staining of FOXA2 and MUC5AC in ALI cultures of human bronchial epithelial cells (16HBE14o-). Partially differentiated, 4-week-old ALI cultures of 16HBE14o- were exposed to PA-derived VOCs for 48 hours to promote differentiation into mucin-secreting goblet cells. Cells were immunolabeled with primary antibodies and detected using secondary antibodies conjugated to Alexa Fluor 594 (red, FOXA2) and Alexa Fluor 488 (cyan-green, MUC5AC). Images were acquired using confocal microscopy and processed into 3D and Z-stack projections. Experiments were independently performed in triplicate. Representative images are shown. **(B, C)** Z-stack quantification of MUC5AC and FOXA2 fluorescence intensity was performed using ImageJ. Densitometry values were normalized to vehicle control (0.1% DMSO in PBS) and presented as mean ± s.e.m. from three independent experiments. Statistical analysis was conducted using one-way ANOVA followed by Tukey’s *post hoc* test; ***p < 0.001, ****p < 0.0001. **(D)** PA-derived VOCs repress FOXA2 and upregulate MUC5AC expression, accompanied by increased IL-17R and IL-17A expression. 16HBE14o- cells were treated for 24 hours with either individual VOCs or a VOC cocktail composed of 2-aminoacetophenone (2-AA; 26 µg/mL), 1-undecene (1UH; 317.5 ng/mL), 2-nonanone (2NA; 22.4 ng/mL), and 1-dodecene (1DH; 9.5 ng/mL). Protein extracts were immunoblotted for MUC5AC, FOXA2, IL-17R, and IL-17A, with GAPDH and histone H3 (H3) as loading controls. **(E)** Densitometry for MUC5AC shown as mean ± s.e.m. Statistical comparisons with PBS control were performed using one-way ANOVA with Tukey’s *post hoc* test; ***p < 0.001.

We first investigated whether PA VOCs in concentrations found in bacterial headspace (7, 9, 11, 42) (**Table 1**) could suppress FOXA2 expression and induce mucin hyper-production in human airway epithelial cells *in vitro*. Because PA secretes multiple VOCs in the diseased airways (8, 9), we also examined a cocktail of 2-AA, 1-undecene, 1-dodecene, and 2-nonanone. Exposure to individual or cocktail of VOCs promoted transdifferentiation of partially differentiated 16HBE14o- cells cultured at the air–liquid interface (ALI) into mucin-secreting goblet cells, with a 3- to 5-fold increase in MUC5AC expression, and a 71% to 78% reduction in FOXA2 expression (**Figures 1A–C**). Consistently, under submerged culture conditions, PA-derived VOCs suppressed FOXA2 and increased MUC5AC expression in 16HBE14o- cells (**Figures 1D, E**).

**Table 1:** Respective VOCs concentrations used in *in vitro* and *in vivo* experiments in this study.

PA-derived VOCs	Dose used in this study	Concentration in bacterial headspace	Reference	Concentration in human samples	Reference
2-aminoacetophenone	26 µg/mL	0.12–192.70 µM	Cox et al. (1979) (42)	Breath: 0–0.1243 ppbv (median 0.0242 ppbv)	Scott-Thomas et al. (2010) (7)
1-dodecene	9.5 ng/mL	1.861–9.5 ppbv	Filipiak et al. (2012) (9)	Not available	—
1-undecene	317.5 ng/mL	3.687–317.5 ppbv	Filipiak et al. (2012) (9)	Sputum: conc. not specified*	Goeminne et al. (2012) (10)
2-nonanone	22.4 ng/mL	1.091–22.4 ppbv	Filipiak et al. (2012) (9)	Sputum: conc. not specified*	Savélev et al. (2011) (8)

The VOCs cocktail consisted of 2-aminoacetophenone (26 µg/mL), 1-undecene (317.5 ng/mL), 2-nonanone (22.4 ng/mL), and 1-dodecene (9.5 ng/mL). Identical concentrations (conc.) were applied in both *in vitro* and *in vivo* experiments. For *in vivo* studies, mice received intranasal instillation of the VOCs mixture once daily for 1 week in a model of chronic airway inflammation. * Absolute VOCs concentrations were not directly specified or directly quantified for absolute concentration in the respective matrices.

### PA-derived VOCs promote transdifferentiation of airway ciliated and club cells into mucus-secreting goblet cells in murine airways

**Figure 2:**
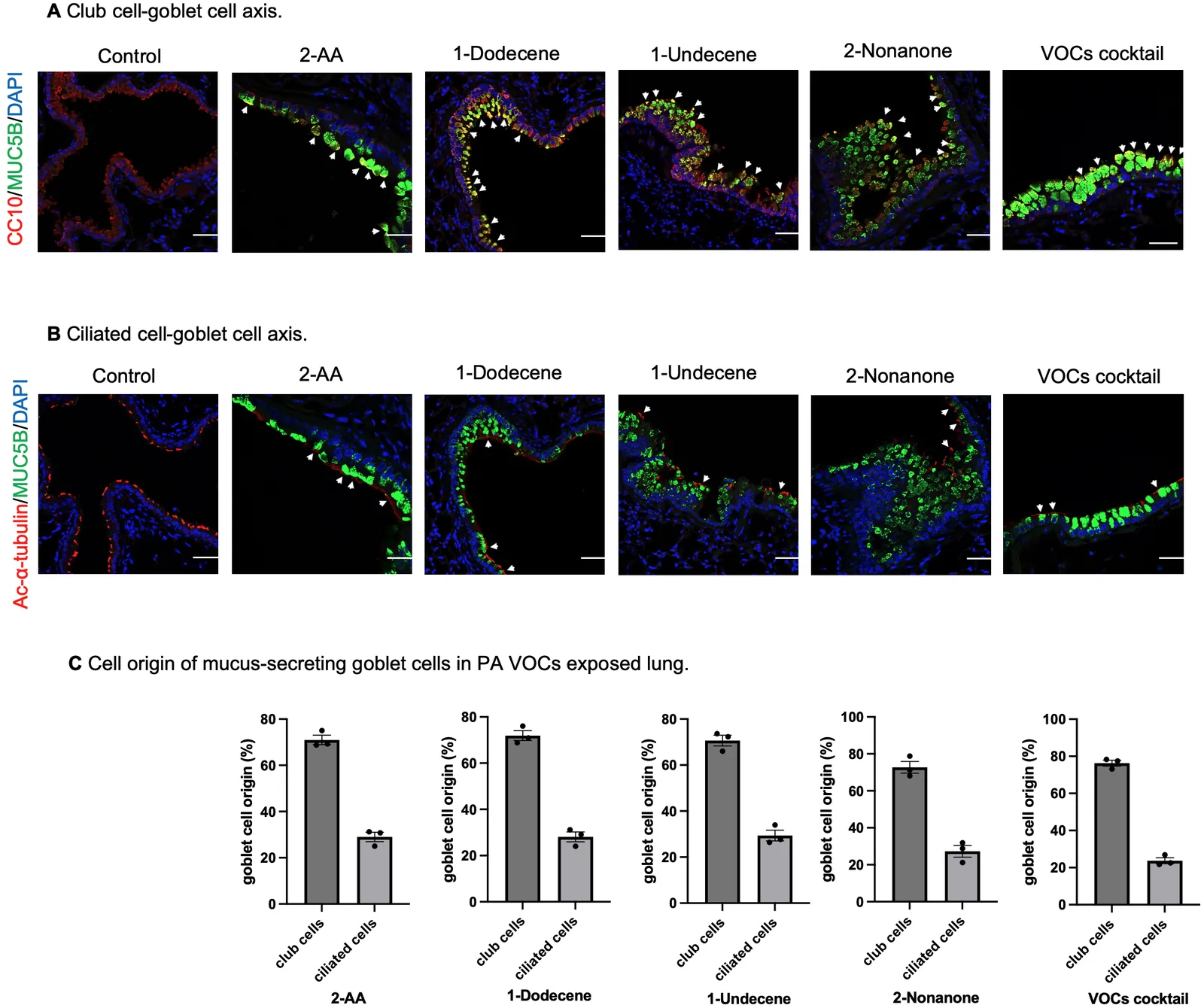
PA-derived VOCs trigger the transdifferentiation of airway ciliated cells and club cells into mucus-secreting goblet cells in murine airways. Lung sections of sex-matched C57BL/6 mice (n = 8) exposed to vehicle control or PA VOCs for 3 weeks were subjected to immunofluorescence staining with specific antibodies against CC10 (club cell marker), acetyl α-tubulin (ciliated cell marker), and MUC5B (goblet cell marker). Lung sections were double-stained with acetyl-α-tubulin/MUC5B or CC10/MUC5B, respectively. Representative images are shown. White arrows indicate double-positive cells. MUC5B was detected with an Alexa Fluor^®^488-conjugated secondary antibody (green). Acetyl-α-tubulin (Ac-α-tubulin) and CC10 were detected using Alexa Fluor^®^647-conjugated secondary antibodies (red). Nuclei were counterstained with DAPI (blue). Scale bar = 50 μm. **(A)** Double immunofluorescence staining of CC10 and MUC5B in PA VOCs-exposed mouse lungs showing club cell-goblet cell axis. **(B)** Double immunofluorescence staining of Ac-α-tubulin and MUC5B in PA VOCs-exposed mouse lungs showing ciliated cell-goblet cell axis. **(C)** Cell origin of mucus-secreting goblet cells in PA VOCs-exposed lung. N = 3 representative airways were counted. Values are presented as mean ± s.e.m. Merged channels are shown; individual fluorescence channels are presented in **Supplementary Figures 1**, **2**.

The airway epithelium layer is a pseudostratified structure composed of multiple cell types, including goblet cells, secretory club cells, ciliated cells, neuroendocrine cells, and basal cells (43). Under homeostatic conditions, goblet cells are sparsely distributed. However, a hallmark of chronic airway diseases is GCM, an epithelial remodeling phenomenon marked by goblet cell transdifferentiation and excessive mucus production. In chronically diseased airways, proinflammatory stimuli often drive the transdifferentiation of multipotent epithelial cells, such as Scgb1a1^+^ secretory club cells and/or FOXJ1^+^ ciliated cells, into mucin-producing goblet cells (43). We analyzed GCM by examining the lineage origins of goblet cells following VOCs exposure. Prolonged exposure to individual or cocktail PA VOCs induced extensive transdifferentiation of club cells into goblet cells, accounting for 70.64%–76.29% of the CC10^+^/MUC5B^+^ population (**Figures 2A, C**, white arrows; **Supplementary Figure 1**). In contrast, a smaller proportion of goblet cells originated from ciliated cells, comprising 23.71%–29.36% of the acetyl-α-tubulin^+^/MUC5B^+^ population (**Figures 2B, C**, white arrows; **Supplementary Figure 2**). These findings align with previous reports identifying secretory club cells as the principal source of GCM in response to allergen challenge (19).

### PA VOCs trigger immune polarization toward M1 macrophages, elevate neutrophil accumulation, and enhance type 17 pro-inflammatory responses in murine lungs

**Figure 3:**
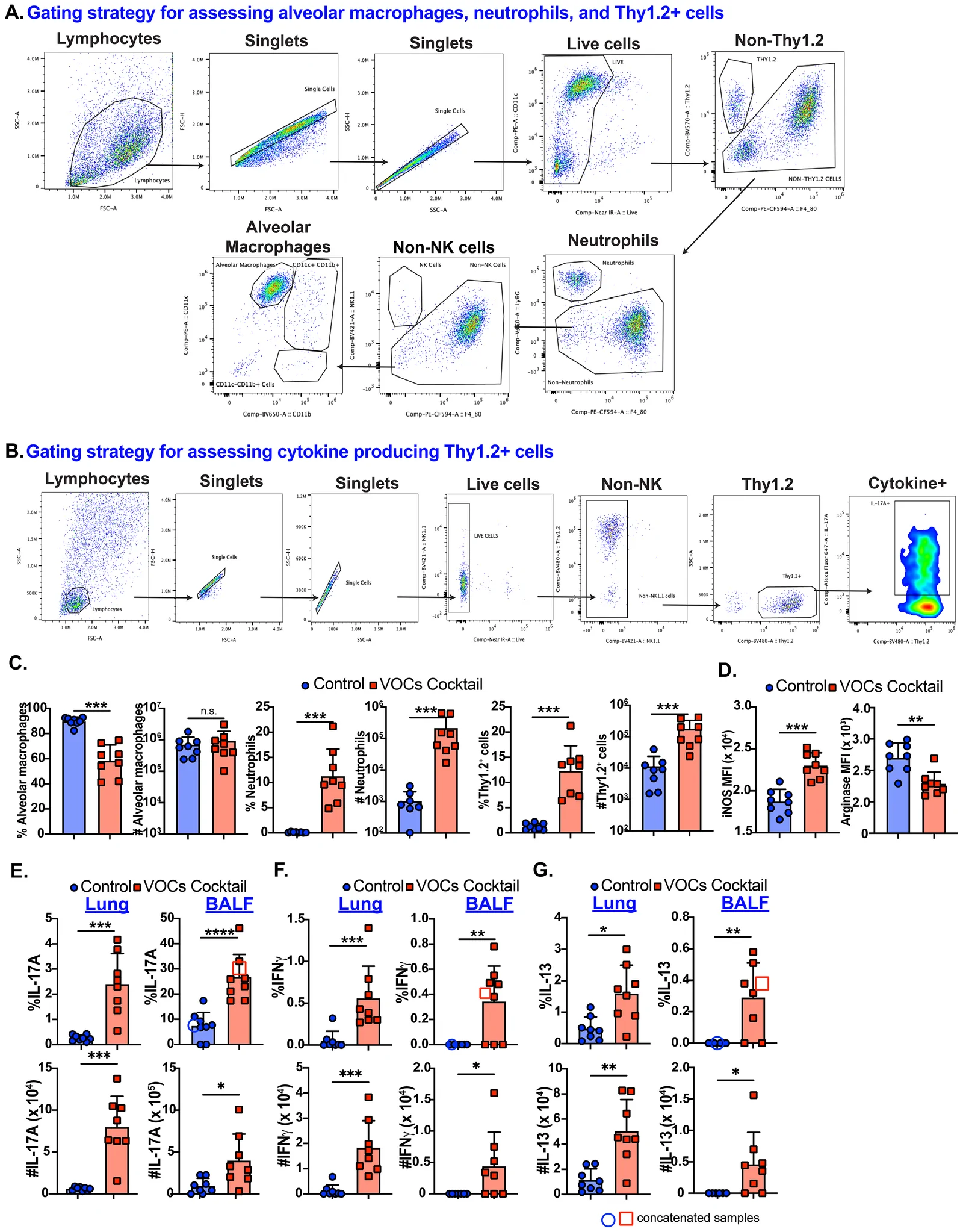
PA VOCs polarize toward M1 macrophages, neutrophils, and type 17 responses that favor mucus hypersecretion. C57BL/6 mice (groups of 8) were intranasally instilled with a vehicle control (0.1% DMSO in PBS) or a VOCs cocktail once daily for 3 weeks. At the end of exposure, single-cell suspensions from BALF and lungs were stained for surface and intracellular markers with fluorochrome-conjugated antibodies and analyzed by flow cytometry. **(A)** Gating strategy to analyze alveolar macrophages, neutrophils, and Thy1.2 cells. **(B)** Gating strategy to analyze cytokine-producing Thy1.2^+^ cells. **(C)** Bar diagrams show the frequencies and absolute numbers of alveolar macrophages (CD45+Ly6G−CD11b−CD11c+F4/80+), neutrophils (CD45+Ly6G+CD11b+), and Thy1.2+ cells among live cells from BALF samples. **(D)** The mean fluorescence intensity (MFI) of iNOS and Arginase that are gated on alveolar macrophages. **(E–G)**. Re-stimulated single cell suspensions were stained for surface markers and intracellular cytokines. Bar diagrams show frequencies and absolute numbers of IL-17A+ **(E)**, IFN-γ+ **(F)**, and IL-13+ **(G)** among live Thy1.2+ lymphocytes in lung/BALF. N = 8 mice/group. Data are expressed as mean ± s.e.m from individual mice values or concatenated data. All statistical analyses were performed using Prism 9.4.1v (GraphPad) software. *p<0.05; **p<0.01; ***p<0.001; ****p<0.0001 by the two-tailed unpaired Student’s t-test.

To determine whether PA VOCs modulate immune signaling that might dysregulate mucus homeostasis, we investigated their effects on type-1, type-2, and type-17, and innate lymphoid cell (ILC)-mediated responses—key immune pathways known to drive GCM and mucus hypersecretion in diseased lungs (26, 44–46). First, we found that, under submerged culture conditions, PA-derived VOCs repressed FOXA2 and upregulated MUC5AC expression in 16HBE14o- cells, with the concomitant increase in IL-17R and IL-17A expression (**Figures 1D, E**), suggesting a potential role for IL-17–mediated signaling in VOCs-induced mucus hypersecretion. We performed flow cytometric analysis on immune cells isolated from BALF and homogenized lungs of C57BL/6 mice after a 3-week exposure to a PA VOCs cocktail following the gating strategies shown in **Figures 3A, B**. Compared to control mice, VOCs-exposed mice exhibited a distinct immune profile in BALF, with significant decrease in the frequency of macrophages, but enhanced neutrophils and Thy1.2^+^ cells significantly accounting for total immune cell populations (**Figure 3C**). Although the frequencies, but not absolute numbers, of alveolar macrophages were reduced in VOCs-exposed mice relative to controls, these macrophages displayed a significant shift toward a proinflammatory M1 phenotype, as indicated by increased iNOS expression and reduced anti-inflammatory M2 phenotype, as indicated by lower Arginase expression (**Figure 3D**). Notably, VOCs exposure significantly increased the frequency of Thy1.2^+^ lymphocytes in both BALF and lung tissue and correlated with elevated levels of IL-17A^+^ lymphocytes (**Figure 3E**). Much smaller, yet statistically significant, increased populations of IFNγ^+^ (Type 1) and IL-13^+^ (Type 2) lymphocytes were also observed following VOCs exposure (**Figures 3F, G**), indicating relatively minor contributions from type 1 and type 2 subsets compared with type 17 responses. Further, we detected an increase in the expression of IL-17 receptor (IL-17R) on the lung epithelia of VOC-exposed mice (**Supplementary Figure 3A**-**C**) as well as in VOC-treated 16HBE14o- cells (**Supplementary Figures 3D**, **E**) compared to their respective controls.

### PA-derived VOCs induce IL-17A expression in murine airway epithelium and in human bronchial epithelial cells

**Figure 4:**
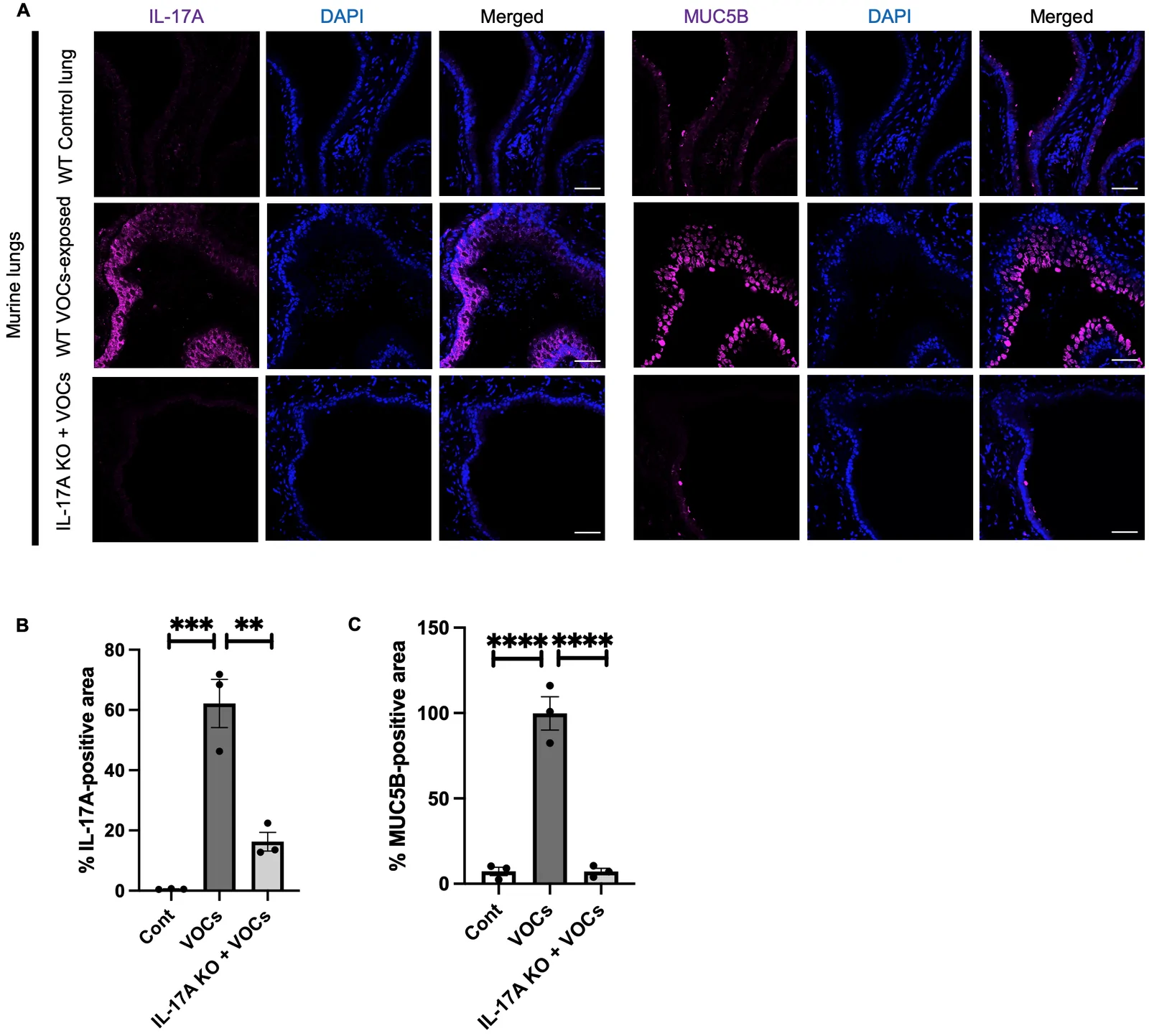
IL-17A expression is upregulated in PA VOCs-exposed wild-type murine airways with mucus overproduction. Lung sections from wild-type (WT) and IL-17A-deficient sex-matched mice (n = 8) exposed to PA VOCs for one week were stained with primary antibodies against IL-17A and the goblet cell-specific marker MUC5B. Nuclei were counterstained with DAPI (blue). Immunolabeling was visualized using secondary antibodies conjugated to Alexa Fluor 647 (magenta, IL-17A and MUC5B). Representative airway sections are shown. Scale bar = 50 μm. **(A)** L-17A expression is upregulated in the airways of PA VOCs-exposed WT mice. **(B, C)** Quantification of IL-17A and MUC5B immunofluorescence in murine airways. N = 3 representative airways were counted. Data are expressed as mean ± s.e.m from individual mice values. The percentage of IL-17A- and MUC5B-positive areas was analyzed using one-way ANOVA followed by Tukey’s *post hoc* test. **p < 0.01, ***p < 0.001, ****p < 0.0001.

The airway epithelium is essential for coordinating both innate and adaptive immune responses in the lungs (47). IL-17A expression was found to be increased in the diseased human airway epithelium; however, the specific role of epithelial-derived IL-17A in the pathogenesis of chronic lung diseases remains incompletely understood (48). To further explore the implications of the airway epithelium-derived IL-17A induced by PA VOCs, we examined IL-17A expression by them *in vivo*, taking advantage of the availability of the IL-17A knockout (KO) mice (49). IL-17A expression was markedly elevated in VOCs-exposed airways of wild-type mice compared with the airways of IL-17A KO mice (**Figures 4A, B**; **Supplementary Figure 4**). Notably, the increased IL-17A expression colocalized to the areas of increased expression of MUC5B in the airways (**Figures 4A, C**; **Supplementary Figure 4**), suggesting that IL-17A plays a role in mucus overproduction. Consistent with this, we also observed elevated IL-17A levels in lung tissues from humans with CF and COPD (**Supplementary Figures 5A, C, D**), as well as in the airways of a COPD dog that died of severe PA infection (**Supplementary Figure 5B, E**).

### Airway epithelium–derived IL-17A promotes VOCs-induced mucus production through an autocrine mechanism

**Figure 5:**
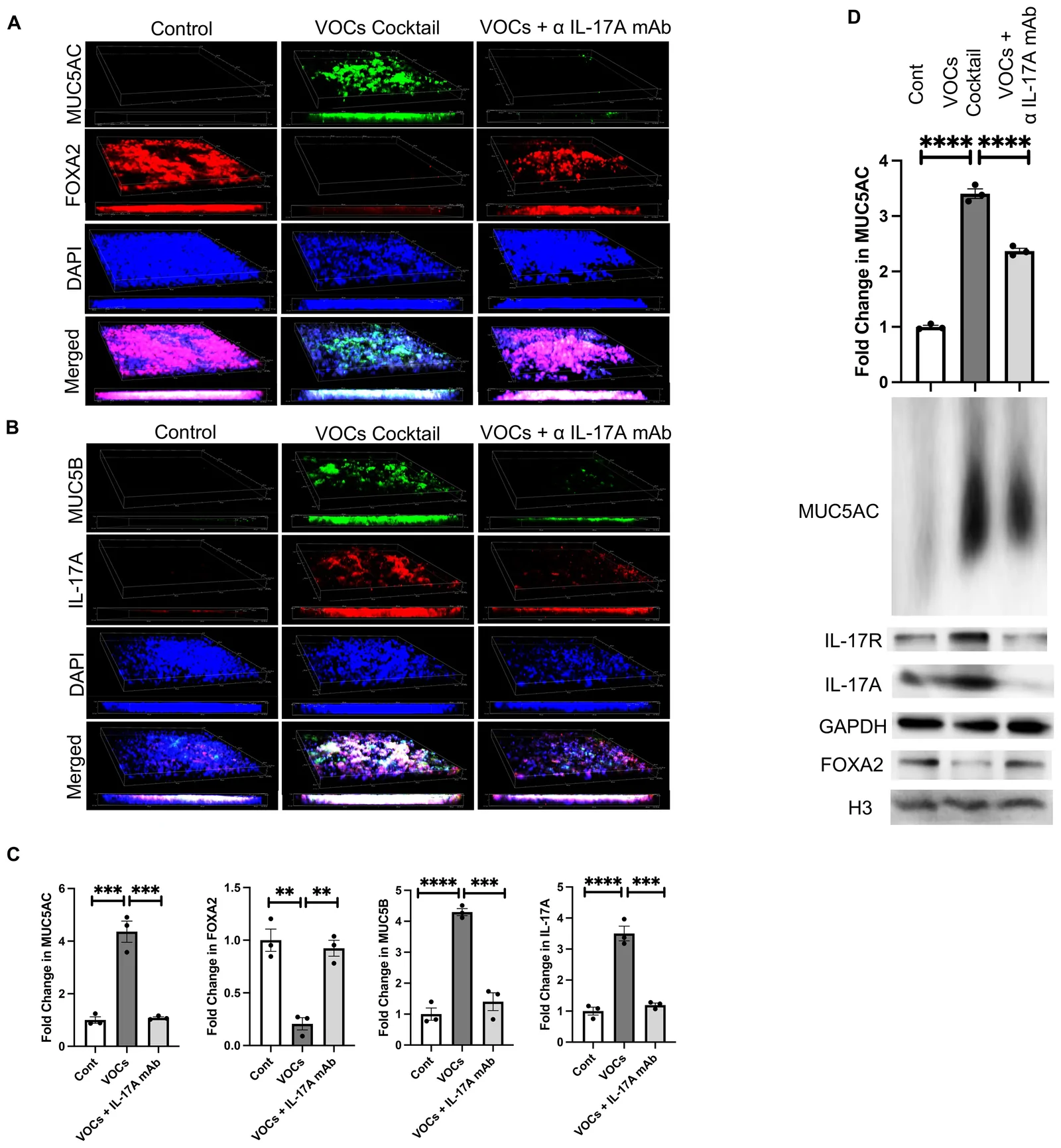
Epithelium-derived IL-17A promotes mucus hypersecretion in an autocrine manner in PA VOCs-exposed ALI and submerged cultures of 16HBE14o^-^ cells. Four-week-old, partially differentiated ALI cultures of human bronchial epithelial cells were potentiated to differentiate into mucin-secreting goblet cells by exposure to PA VOCs cocktail for 48 hours in the presence or absence of the IL-17A neutralizing antibody (IL-17A mAb, 12047-M237, 1.0 μg/ml, one hour prior to VOCs exposure). Cells were labeled with specific primary antibodies and visualized using secondary antibodies conjugated to Alexa Fluor 594 (red, FOXA2 and IL-17A) or Alexa Fluor 488 (cyan-green, MUC5AC and MUC5B), followed by 3D and z-stack confocal microscopy. Experiments were independently repeated in triplicate. Representative images are shown. **(A)** ALI culture of 16HBE14o^-^ cells double-stained for MUC5AC and FOXA2. **(B)** ALI culture of 16HBE14o^-^ cells double-stained for MUC5B and IL-17A. **(C)** Z-stack image quantification of MUC5AC, FOXA2, MUC5B, and IL-17A expression using ImageJ. Densitometry values were normalized to vehicle-treated controls (0.1% DMSO in PBS; Cont). Data are presented as mean ± s.e.m. from three experiments. Statistical analysis was performed using one-way ANOVA followed by Tukey’s *post hoc* test. **p < 0.01, ***p < 0.001, ****p < 0.0001. **(D)** IL-17/IL-17RA signaling promotes MUC5AC expression during PA VOCs-induced mucus hypersecretion. Pretreatment with IL-17A mAb restored FOXA2 expression and attenuated MUC5AC production in 16HBE14o^-^ cells upon VOCs exposure. Protein expression was analyzed via immunoblotting using antibodies against IL-17R, IL-17A, MUC5AC, and FOXA2. GAPDH and histone H3 served as loading controls. Densitometric analysis of MUC5AC bands was normalized to GAPDH. Data are shown as mean ± s.e.m. ****p < 0.0001 by one-way ANOVA with Tukey’s *post hoc* test.

To authenticate the results in **Figure 4** and **Supplementary Figure 4**, we performed *in vitro* experiments using ALI cultures. Exposure to VOCs enhanced the IL-17A expression in ALI cultures of 16HBE14o^-^ cells, along with elevated expression of MUC5AC and MUC5B, and a corresponding decrease in FOXA2 (**Figures 5A–C**). Similar results were observed in submerged cultures (**Figure 5D**). Together, these results suggest that airway epithelial cells could be an additional significant source of IL-17A upon exposure to PA VOCs and support a functional role for epithelial-derived IL-17A in driving GCM and mucus overproduction in chronically diseased airways.

Given that PA VOCs-exposed airway epithelial cells not only produce IL-17A but also express its receptor, we hypothesized that epithelial-derived IL-17A may act in an autocrine manner—binding to the IL-17RA/IL-17RC receptor complex on airway cells—to amplify inflammatory signaling and promote mucus production. To address this, we employed an IL-17A–neutralizing antibody (IL-17A mAb, 12047-M237, 1.0 μg/ml) to deplete IL-17A activity in both ALI and submerged cultures of 16HBE14o^-^ cells. Neutralization of IL-17A resulted in a marked suppression of MUC5AC expression and partial restoration of FOXA2 levels in VOCs-challenged cultures (**Figures 5A–D**; **Supplementary Figures 6**, **7**). These findings suggest that following the exposure of PA VOCs, airway epithelial cells produce IL-17A, which can act in an autocrine fashion to drive FOXA2 repression and mucus hypersecretion in the respiratory epithelium.

### IL-17A ablated mice are more resistant to PA VOCs-mediated induction of GCM and mucus hypersecretion

**Figure 6:**
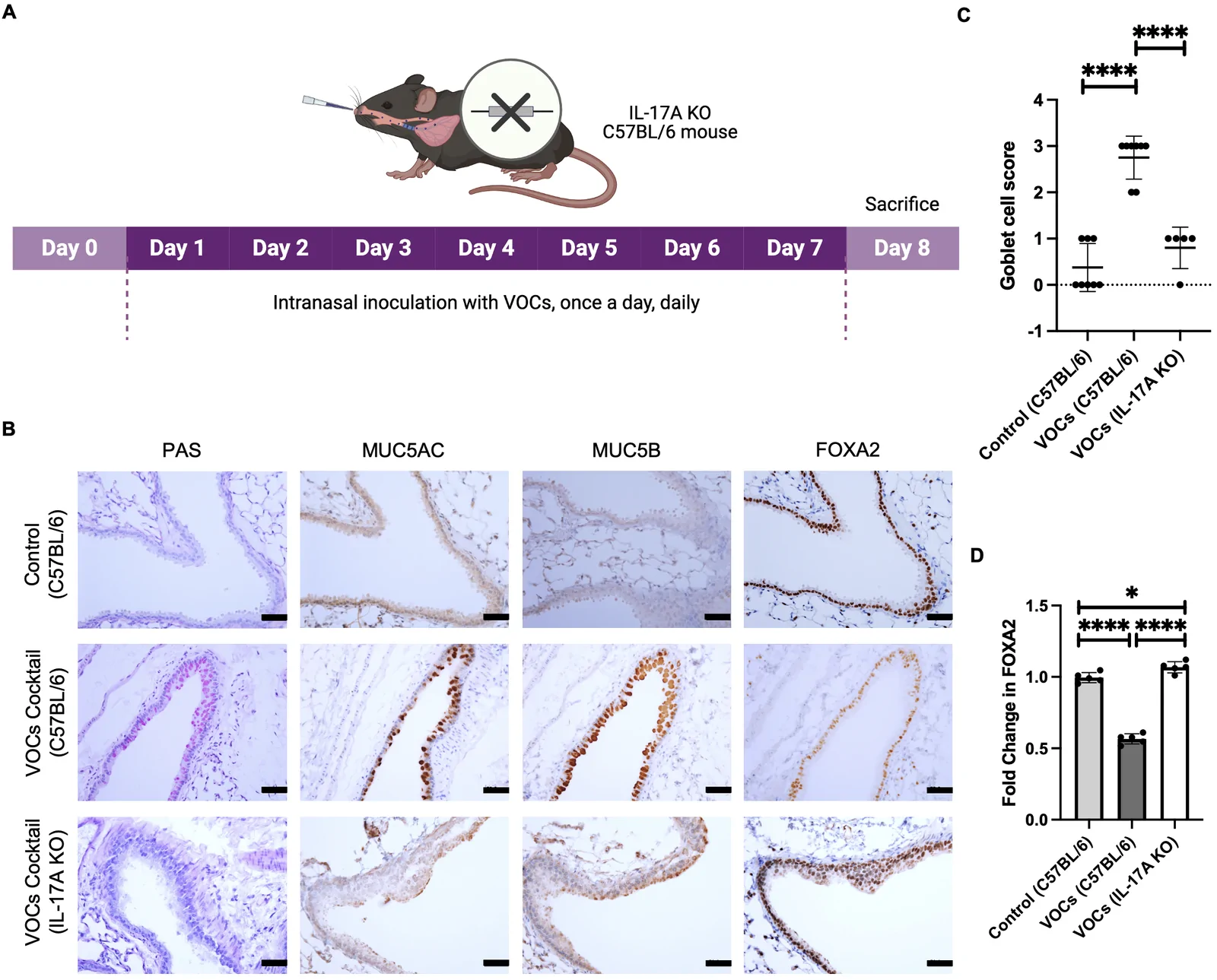
IL-17A KO mice are resistant to PA VOCs-induced GCM and mucus hypersecretion. **(A)** Schematic of experimental design. Sex-matched C57BL/6 or IL-17A KO mice (groups of 8) were exposed to PBS or a VOCs cocktail once daily for 1 week. Lungs were processed for PAS staining and for IHC using specific antibodies against MUC5AC, MUC5B, and FOXA2. **(B)** Representative PAS and IHC-stained lung sections. **(C)** Goblet cell scores in PAS-stained airways. **(D)** FOXA2 expression as quantified by ImageJ. The degree of PAS-stained mucin expression was quantified using a 4-tier grading system as described in the Methods section in a blinded fashion. N = 5–8 bronchioles were counted. Scale bar = 50 μm. Data are shown as mean ± s.e.m.; statistical comparisons were performed using one-way ANOVA followed by Tukey’s *post hoc* test, with significance levels indicated as *p < 0.05, **p < 0.01, ***p < 0.001, and ****p < 0.0001. Created in BioRender. Kuo, S. H. (2026) https://BioRender.com/31jt1wm.

To test the hypothesis that a heightened IL-17A expression contributed to GCM and mucus hypersecretion in VOCs-exposed murine airways, we performed selective depletion of IL-17A in VOCs-exposed mice. Initial *in vivo* attempts to block IL-17A with a neutralizing antibody were unsuccessful (data not shown), possibly due to incomplete neutralization and insufficient effective concentration in the lung. In addition, while IL-17A is regarded as the major player, other IL-17 family cytokines may also contribute to airway inflammation and mucus production, and complicate the IL-17A blockage alone. For instance, IL-17E (IL-25) induces type 2 inflammatory responses such as IL-4, IL-5, and IL-13 signaling, which are directly associated with GCM and increased mucin production (e.g., MUC5AC) (50). Also, IL-17F is involved in mucus hypersecretion, mainly by promoting goblet cell hyperplasia and metaplasia (51). To overcome this, we used IL-17A KO mice (49) to define the role of IL-17A in PA VOCs-mediated GCM (**Figure 6A**). In IL-17A KO mice exposed to PA VOCs, there was a significant reduction in the number of PAS-stained goblet cells and the expression levels of MUC5AC and MUC5B, with a low goblet cell score of 0.8 ± 0.4, compared with a higher goblet cell score of 2.8 ± 0.4 in C57BL/6 wild-type control mice (**Figures 6B, C**). Moreover, nuclear FOXA2 level was not reduced (**Figures 6B, D**). These results suggest that PA VOCs-induced IL-17A represses the expression of FOXA2 and triggers GCM and excessive mucus production after PA VOCs exposure. Further, the strong phenotype observed with IL-17A KO mice suggests that IL-17A is a major player among the other IL-17 family cytokines in driving PA VOCs-mediated GCM, and an incomplete neutralization of IL-17A is likely the reason for the lack-of-effect phenotype in our preliminary studies.

### Macrophage depletion restores FOXA2 expression and mitigates mucus overproduction in mouse airways exposed to PA VOCs

**Figure 7:**
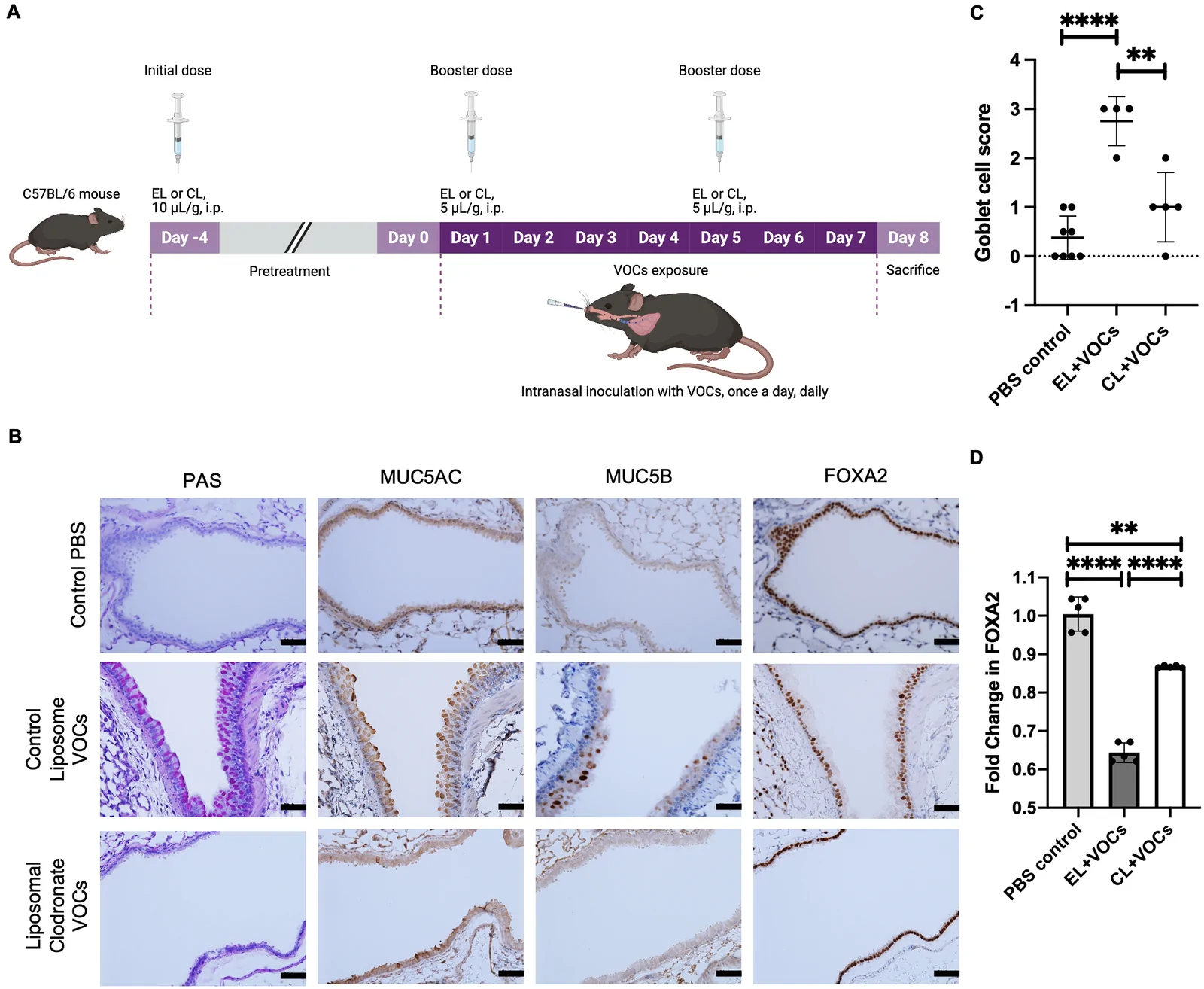
Depletion of macrophages restores FOXA2 nuclear expression and ameliorates mucus hypersecretion in mouse airways exposed to PA VOCs. **(A)** Schematic of experimental design. Sex-matched C57BL/6 (groups of 8) were intraperitoneally injected with 10 μL/g mouse weight of clodronate-containing liposome **(CL)** or empty liposome (EL) on Day -4 before the first VOC exposure, and then at 5 μL/g mouse weight on Days 1 and 5. The VOCs cocktail was administered once daily. On Day 8, lungs were processed for PAS staining and for IHC staining using specific antibodies against MUC5AC, MUC5B, and FOXA2. **(B)** Representative PAS and IHC-stained lung sections. **(C)** Goblet cell scores in PAS-stained airways. **(D)** FOXA2 expression as quantified by ImageJ. N = 5–8 bronchioles were counted. Scale bar = 50 μm. Data are shown as mean ± s.e.m.; statistical comparisons were performed using one-way ANOVA followed by Tukey’s *post hoc* test, with significance levels indicated as *p < 0.05, **p < 0.01, ***p < 0.001, and ****p < 0.0001. Created in BioRender. Kuo, S. H. (2026) https://BioRender.com/h2ad9fk.

We next examined the role of macrophages in promoting mucus hypersecretion in response to PA-derived VOCs. To deplete macrophages *in vivo*, mice were administered clodronate liposomes (CL), and control groups received empty liposomes (EL) (FormuMax Scientific Inc., #F70101C-N and F70101-N, respectively) via intraperitoneal injection according to the manufacturer’s instructions (**Figure 7A**). Systemic depletion of macrophages attenuated GCM and decreased the expression of MUC5AC and MUC5B in response to the PA VOCs challenge, reflected by a lower goblet cell score of 1.0 ± 0.6 (**Figures 7B, C**). In contrast, a marked increase in goblet cells and upregulated MUC5AC and MUC5B were found following the exposure to PA VOCs in control mice receiving empty liposomes (2.8 ± 0.4) (**Figures 7B, C**). There was a significant difference in goblet cell scores between the clodronate and control liposome administered groups exposed to PA VOCs (p = 0.004). Further, nuclear FOXA2 level was restored to 87% in the CL-treated mice (**Figures 7B, D**), indicating that FOXA2 expression and its suppressive function against GCM were well recovered. Thus, these data support the contribution of PA VOCs-activated macrophages in the disruption of airway mucus homeostasis.

### Neutrophil depletion partially restores FOXA2 expression and reduces mucus hypersecretion in PA VOCs-exposed mouse airways

**Figure 8:**
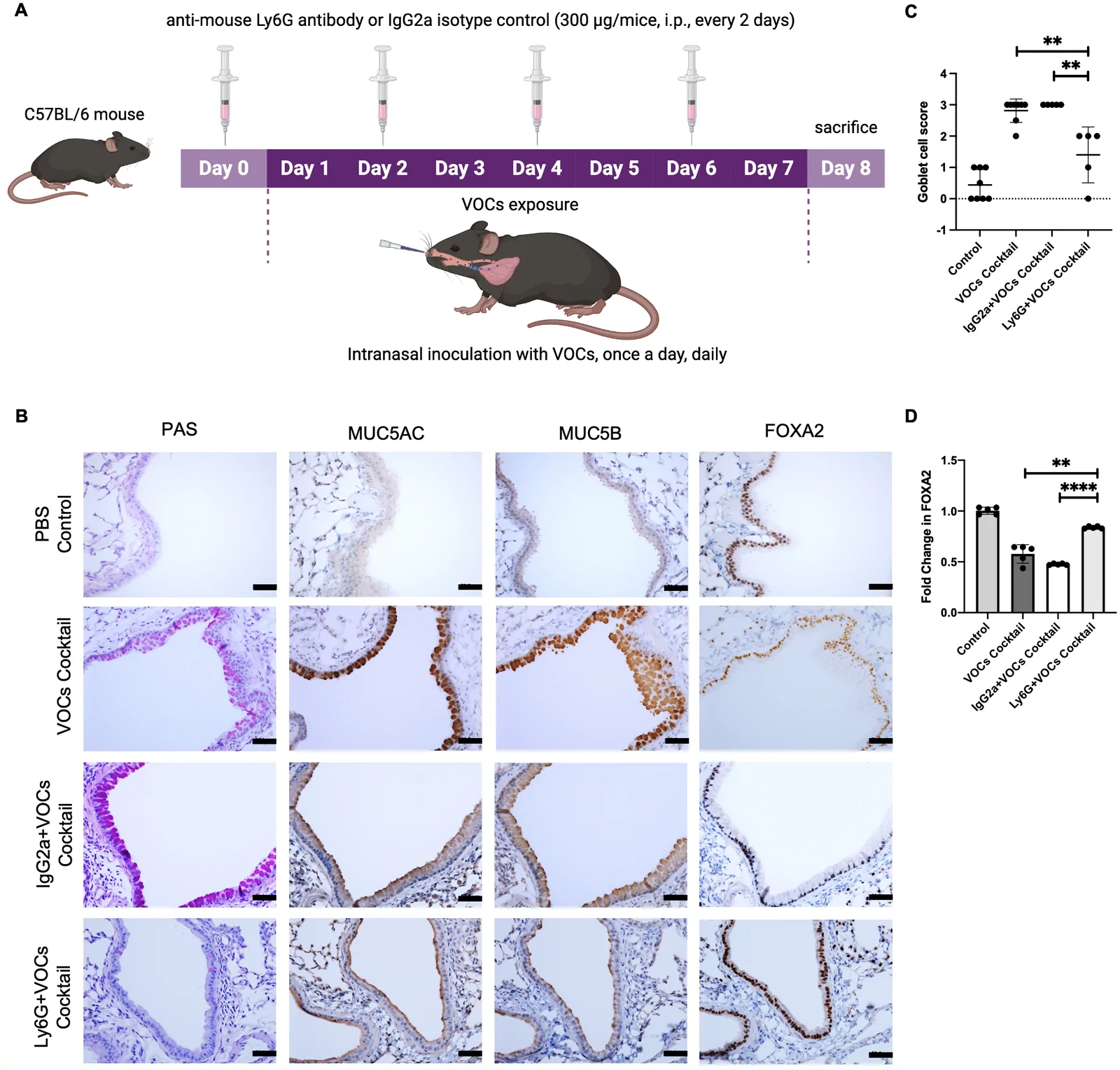
Depletion of neutrophils partially restores FOXA2 expression to attenuate mucus hypersecretion in PA VOCs-exposed mouse airways. **(A)** Schematic of experimental design. Sex-matched C57BL/6 mice (groups of 8) were depleted of systemic neutrophils with 300 μg rat anti-mouse Ly6G (Clone 1A8) or with rat IgG2a isotype control (Clone 2A3) by intraperitoneal injection on Day 0, and on Days 2, 4, and 6, one hour before daily exposure to VOCs. Mouse lungs were analyzed on Day 8. Mouse lungs were processed for PAS staining and for IHC using specific antibodies against MUC5AC, MUC5B, and FOXA2. **(B)** Representative PAS and IHC-stained lung sections. **(C)** Goblet cell scores in PAS-stained airways. **(D)** FOXA2 expression as quantified by ImageJ. N = 5–8 bronchioles were counted. Scale bar = 50 μm. Data are shown as mean ± s.e.m.; statistical comparisons were performed using one-way ANOVA followed by Tukey’s *post hoc* test, with significance levels indicated as *p < 0.05, **p < 0.01, ***p < 0.001, and ****p < 0.0001. Created in BioRender. Kuo, S. H. (2026) https://BioRender.com/smvb7ry.

Since we found significantly increased neutrophil infiltration (**Figure 3C**), we set out to determine their role in the induction of GCM and increased mucus expression. We systemically depleted neutrophils using anti-mouse Ly6G mAb (intraperitoneally administered, Clone 1A8; BioXCell) (**Figure 8A**) in PA VOCs-exposed mice. Neutrophil depletion resulted in a moderate reduction of GCM, with a goblet cell score of 1.4 ± 0.8 (**Figures 8B, C**) and a significant decrease in mucin production in PA VOCs-exposed mice compared with the control antibody (IgG2a isotype, Clone 2A3; BioXCell) administered group exposed to VOCs (**Figures 8B, C**). Furthermore, nuclear expression of FOXA2 was restored to 84% in the neutrophil-depleted mice (**Figures 8B, D**). Thus, neutrophils also contribute to the modulation of FOXA2 expression and the regulation of mucin production in PA VOCs-exposed airways.

## Discussion

**Figure 9:**
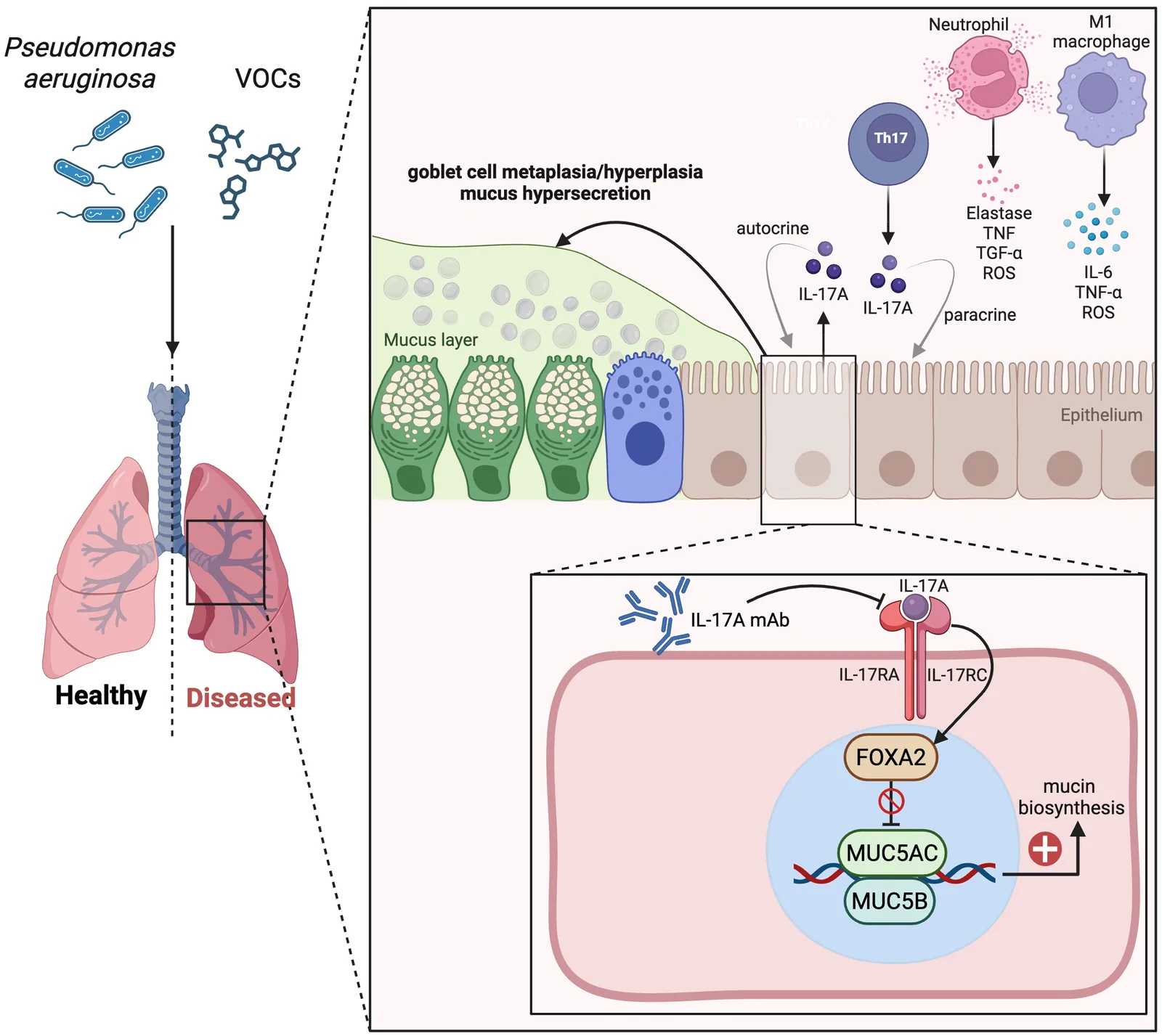
Graphical abstract. In summary, our findings indicate that PA-derived VOCs are capable of inducing mucus overproduction through a shift toward a proinflammatory pulmonary environment, characterized by elevated IL-17A expression, activation of M1 macrophages, and enhanced infiltration of neutrophils. Epithelial-derived IL-17A acts in an autocrine manner, alongside paracrine signaling from IL-17A–producing type 17 lymphocytes, to suppress FOXA2 expression and regulate mucin hypersecretion. Created in BioRender. Kuo, S. H. (2026) https://BioRender.com/rxgrqlp.

We showed that chronic exposure to PA-derived VOCs—2-AA, 1-dodecene, 1-undecene, 2-nonanone, and a cocktail of these VOCs—causes GCM and mucus hypersecretion in mice. VOCs exposure increases proinflammatory response in the mouse lung, evidenced by enrichment of M1 macrophages, neutrophils, and type 17 lymphocytes in BAL samples—all of which have been implicated in GCM and mucus hypersecretion (52–55). Conditional removal of macrophages or neutrophils restored the expression of FOXA2, an important transcriptional repressor of mucin genes, to suppress mucin production in VOCs-exposed airways. Similarly, IL-17A KO mice were more resistant to PA VOCs-induced inhibition of FOXA2 expression and increased mucus production. Collectively, these results indicate that PA VOCs play a critical role in mucus dysregulation in chronically diseased lungs by inducing M1 macrophage polarization, neutrophilic inflammation, and type 17-mediated immune responses that inhibit FOXA2 expression, as summarized in **Figure 9**.

Previous studies have documented that synergistic interactions exist between M1 macrophages, neutrophils, and pro-inflammatory Th17^+^ cells (56–58). M1 macrophages produce IL-6 and IL-23 that promote the differentiation of Th17 cells by upregulation of the transcription factor RAR-Related Orphan Receptor Gamma (RORγt) (59). Notably, RORγt skews towards the development of IL-17^+^/GM-CSF^+^ CD4^+^ T cells, which further reinforces M1 macrophage differentiation (56, 57). Similarly, neutrophils can prime the differentiation and the activation of pro-inflammatory M1 macrophages (58). Finally, IL-17A is involved in the activation of neutrophils, well-known primary producers of reactive oxygen species (ROS), which, in turn, could induce EGFR signaling (60) and increase mucus biosynthesis. The intricate interplay among these components establishes a positive proinflammatory feedback circuit that amplifies mucus hypersecretion induced by PA VOCs. This conclusion is supported by our findings that selective depletion of either one of the three immune components attenuates the ability of PA VOCs to disrupt airway mucus homeostasis.

Increased mucus secretion in the airways is an important pathophysiological element of CF, COPD, bronchiectasis, asthma, and other chronic inflammatory disorders of the airways (1, 2). Recent investigations have shown that inflammation, oxidative stress, and airway mucosal microbiome are fundamental to mucus pathology in diseased lungs and are closely associated with clinical outcomes. Among the GCM and mucus-inducing proinflammatory mediators, the IL-17 family of cytokines plays a critical role in inflammatory airway autoimmune and allergic diseases, and infections (61). Among the six members of the IL-17 family—IL-17A, -17B, -17C, -17D, -17E, and -17F,—IL-17A has been particularly linked to the control of PA chronic infection (62) and the progression of CF (63) and COPD (64). IL-17A enhances mucus production via multiple pathways. For example, IL-17A acts through IL-5 and IL-6 to induce the expression of *MUC5B* and *MUC5AC* genes in tracheobronchial epithelial cells *in vitro* (45). Similarly, IL-17A produced by epithelial cells contributes to inflammation and augmented mucus production upon exposure to cigarette smoke (65). In agreement with our observations that PA VOCs-induced IL-17 response drives mucus hypersecretion, IL-17A expression was found to positively correlate with MUC5AC overexpression in a mouse model of asthma following respiratory syncytial virus infection (66).

The airway epithelium is important in regulating both lung innate and adaptive immunity. Overexpression of IL-17A is a feature of CF lung disease and correlates with neutrophil inflammation, epithelial injury, and worsening respiratory function (67, 68). Upon binding to the IL-17RA/IL-17RC receptor complex, IL-17A triggers epithelial cells to release proinflammatory cytokines, thereby enhancing the inflammatory milieu and mucin production (69, 70). Recent studies have demonstrated that in addition to Th17 cells, IL-17A is produced in inflamed lungs by innate immune cells and structural epithelial cells (65). Importantly, increased IL-17A expression has been observed in airway epithelial cells of COPD patients—both *in vivo* (bronchial biopsies) and *in vitro* following treatment with diesel exhaust particles or cigarette smoke (65, 71, 72). Similarly, we found increased IL-17A expression in the airway epithelium of human CF and COPD airway epithelium and in canine COPD airways with PA infection. Furthermore, we show that IL-17A expression is significantly enhanced in mouse airway epithelial cells following exposure to PA VOCs, supporting that diseased airway epithelium is a significant source of IL-17A during inflammation. Selective antibody-mediated neutralization of IL-17A in both submerged and ALI culture models attenuated mucin overexpression, suggesting that epithelial-derived IL-17A, acting in an autocrine manner, operates in parallel with paracrine signaling from IL-17A secreted by type 17 lymphocytes to regulate mucin expression. Our data establish a wider role for IL-17A in regulating the overproduction of mucus by GCM and suppressing FOXA2 expression.

IL-17A-dependent immunity is critical for the host’s defense against both acute and chronic infections by facilitating neutrophil recruitment and clearance of extracellular respiratory bacterial pathogens (e.g., *Streptococcus pneumoniae*, *Klebsiella pneumoniae*, PA), with innate lymphoid cells being the predominant source of IL-17A during the early phases (62, 73). However, as discussed above, elevated levels of IL-17A are found in clinical samples that correlate with neutrophilic inflammation, lung damage, and lung function decline in CF and COPD patients, hence representing a potential risk factor for PA infection or PA-infection induced disease exacerbation (62, 65, 67, 68, 73, 74). Accordingly, neutralization of IL-17A and knockout mice deficient in IL-17A and IL-17RA are protected from PA-mediated fatal pneumonia (74–76). Altogether, these observations suggest that while IL-17A is crucial for initial host defense, its sustained expression may exacerbate PA infections. The polarization of the immune response towards IL-17A-dominant immunity by PA VOCs, therefore, may exacerbate PA disease pathogenesis and progression.

Our data indicate that PA VOCs induced M1 macrophages play an important role in driving mucus hypersecretion in mouse lungs. The classically activated M1 macrophages are pro-inflammatory and secrete a plethora of cytokines, including IL-6, IL-1β, TNF, TGF-α, and MCP-1, as well as generating ROS and nitric oxide (NO). ROS induces a variety of signaling cascades that augment mucin gene expression in airway epithelial cells and hence result in increased mucus production (4, 24, 25, 29–32, 35). Similarly, NO regulates mucin synthesis, often in concert with other inflammatory mediators (77). Andelid et al. demonstrate that chronically activated and partially steroid-resistant sputum macrophages perpetuate TNFα-dependent signals that drive mucus hyperproduction in bronchial epithelial cells in chronic bronchitis (52). Moreover, MCP-1 recruits macrophages to the airways and further stimulates the expression of proteases that impair mucociliary clearance and promote mucostasis (78). CF airways are characterized by an increased protease-antiprotease imbalance, resulting in bronchiectasis and a progressive decline in lung function. Collectively, the byproducts from M1 macrophages substantially influence mucus dysregulation and lung pathophysiology, thereby aggravating the severity of chronic airway diseases.

PA VOCs induce a large influx of neutrophils in murine airways. Activated neutrophils secrete TNF and release ROS, which activate the pro-GCM EGFR signaling in airway epithelial cells (55). Also, neutrophil elastase (NE) exhibits dual actions in dysregulating airway mucus by inducing robust goblet cell degranulation and concurrently cleaving pro-TGF-α into its mature form that activates EGFR-dependent mucin overexpression in a ligand-dependent manner (55). These interconnected biological processes further explain how PA VOCs-induced neutrophilia promotes GCM and mucus hypersecretion.

A major limitation in the field is the scarcity of direct *in vivo* measurements of absolute PA-derived VOCs concentrations within the human airway; therefore, the VOCs concentrations used in this study were selected based on previously reported bacterial headspace measurements (9, 42). Although volatilome studies using analytical platforms such as GC-MS/SPME, SIFT-MS, and PTR-MS have identified PA-associated VOCs in respiratory specimens from CF patients (6–8, 12, 79–83), quantitative assessment remains limited because most studies lack internal standards necessary for determining absolute compound concentrations. The currently reported human concentration of 2-AA was derived from exhaled breath measurements (0.0242 ng/mL) (7), which likely underestimates concentrations within the airway microenvironment because exhaled VOCs represent diluted end-expiratory gas and are additionally subject to evaporation and sample loss during specimen collection and processing. In contrast, sputum or sinus mucus may better reflect local airway surface liquid concentrations; however, studies evaluating 2-AA in BALF (83), sputum (8, 10), or sinus mucus (84) report only relative abundance values, and without internal standards, precluding determination of physiologically relevant absolute concentrations. Evidence from HCN, another PA-derived VOCs for which both breath and sputum concentrations have been reported, illustrates a concentration discrepancy. Whereas exhaled breath HCN concentrations are approximately 0.0143 ng/mL (80), sputum concentrations range from 3,514–10,812 ng/mL (5, 83), representing a 10^5^–10^6^-fold difference between compartments. Similarly, the approximately 6-log discrepancy between exhaled breath-derived 2-AA concentrations (0.0242 ng/mL) reported in the literature and the bacterial headspace-derived concentrations (26 μg/mL) used in our study suggests that breath measurements may substantially underestimate local airway exposure, and that the higher concentrations detected in bacterial headspace may still be physiologically plausible within sputum and/or the alveolar microenvironment during chronic PA colonization. Notably, the impaired mucociliary clearance and airflow that are characteristic of diseased airways may trap and further concentrate VOCs in the retained mucus, potentially amplifying local exposure. Importantly, we have also observed MUC5AC induction and FOXA2 degradation in ALI-cultured 16HBE cells exposed to the substantially lower breath-reported concentration of 2-AA (85), further supporting the biological relevance and robustness of our findings across a broad exposure range.

Another consideration regarding the translational relevance of our mouse model is that published measurements of bacterial VOCs have been obtained primarily from the gas phase, whereas in the present study, VOCs exposure was delivered intranasally as an aqueous solution in 0.1% DMSO-PBS. This is biologically relevant, as the airway mucus is approximately 95–98% water, with the remaining components being mucins, non-mucin proteins, lipids, salts, and cellular debris (1). In PA infection, excessive mucus accumulation obstructs the airways and provides an aqueous medium in which VOCs can dissolve and partition. Thus, delivery of VOCs in an aqueous vehicle may more closely mimic the *in vivo* conditions during infection than VOCs exposure in the gas phase alone, as it models the interaction of these compounds with the mucus layer and the adjacent airway epithelium.

Because of the strong inter-regulatory network between M1 macrophages which promote IL-17A differentiation through the secretion of IL-6 and IL-23; IL-17A plays a crucial role in host defense against both fungal and bacterial infection at mucosal surfaces (86–88), including its synergistic with TNFα in recruiting neutrophils, a major arm of the innate immunity system against microbial infection (89, 90), global inhibition of these immune components to attenuate mucus pathology caused by the PA VOCs will be impractical and counterproductive. Airway-specific neutralization of the pathogenic effects of M1 macrophages, and autocrine IL-17A secretion by airway epithelial cells and paracrine secretion by infiltrating Th17 cells will attenuate infiltration of neutrophils and proinflammatory damage (e.g., by tissue-damaging neutrophil elastase and ROS), and restore FOXA2 function to alleviate mucus pathology. Another promising approach will be to inhibit VOCs biosynthesis by aerosolizing N-Aryl Malonamides targeting the PA master regulator MvfR (PqsR) (91) in the airway epithelium. MvfR activates the expression of many VOCs biosynthetic genes, including 2-AA and HCN.

In summary, our findings indicate that PA-derived VOCs are capable of inducing mucus overproduction through a shift toward a proinflammatory pulmonary environment, characterized by elevated IL-17A expression, the activation of M1 macrophages, and enhanced infiltration of neutrophils. These immune components operate in a synergistic, feed-forward loop to intensify mucus hypersecretion and amplify airway inflammation, establishing a self-reinforcing circuit that contributes to disease progression. Our study demonstrates the immunomodulatory effects of PA-derived VOCs on host airway responses, revealing their pivotal role in promoting mucus hypersecretion and exacerbating chronic respiratory diseases.

## Data Availability

The raw data supporting the conclusions of this article will be made available by the authors, without undue reservation.
