# Supplementary material for: Pseudomonas aeruginosa-derived volatile organic compounds modulate host immunity to disrupt airway mucus homeostasis
Source: Front Immunol. 2026 Aug 26;17:1823251. doi: 10.3389/fimmu.2026.1823251 (PMC13553463; doi:10.3389/fimmu.2026.1823251)
Supplement: Supplementary file 1 [file DataSheet1.pdf]

Pseudomonas aeruginosa-derived volatile organic compounds modulate host immunity to disrupt airway mucus homeostasis

Shanny Hsuan Kuo[1,2], Jaishree Sharma[1,3], Cong Wu[1], Miranda D. Vieson[1,4], Beata Kosmider[5], Scott H. Randell[6], Som G. Nanjappa[1*], and Gee W. Lau[1*]

[1] Department of Pathobiology, College of Veterinary Medicine, University of Illinois Urbana-Champaign, Urbana, IL, USA.

[2] Department of Comparative Medicine, School of Medicine, University of Washington, Seattle, WA, USA.

[3] Fred Hutchinson Cancer Center, Seattle, WA, USA.

[4] Veterinary Diagnostic Laboratory, College of Veterinary Medicine, University of Illinois Urbana-Champaign, Urbana, IL, USA.

[5] Center for Inflammation and Lung Research, Department of Microbiology, Immunology and Inflammation, Lewis Katz School of Medicine, Temple University, Philadelphia, PA, USA.

[6] Marsico Lung Institute/Cystic Fibrosis Center, School of Medicine, University of North Carolina at Chapel Hill, Chapel Hill, NC, USA.

[*]**For correspondence:** nanjappa@illinois.edu; geelau@illinois.edu

Supplementary Methods and Figures

Methods

Concise experimental methods are provided in the main text. A detailed description of experimental methods is provided below.

Chemicals, reagents, and antibodies. VOCs, including 2-AA (W390607), 1-Dodecene (D221600), 1-Undecene (242527), and 2-Nonanone (108731), were purchased from Sigma-Aldrich. Compounds were first suspended in DMSO and then serially diluted to desired concentrations in sterile PBS (final DMSO concentration < 0.1%). Antibodies utilized in this study include: Santa Cruz Biotechnology (FOXA2, sc-101060; MUC5AC, sc-21701; MUC5B, sc-20119; IL-17R, sc-376374; IL-17A, 740021M and PA5-114455; CC10, sc-365992); Invitrogen (acetyl-α tubulin, 6-11B-1); Cell Signaling Technology (FOXA2 D56D6); Bioss Antibodies (MUC5AC bs-7166R); and Novus Biologicals (MUC5B NBPI-92151). IL-17A neutralizing antibody (12047-M237) was purchased from Sino Biological, Inc. Antibodies were diluted in 5% bovine serum albumin, used at dilutions recommended by manufacturers, and optimized as needed.

Healthy, CF, and COPD human and canine lung tissues. De-identified healthy and CF-diseased or COPD-diseased lung tissue sections from deidentified donors were provided by Professor Scott Randell (University of North Carolina) and Professor Beata Kosmider (Temple University), respectively. The diseased lung sections demonstrated confirmed clinical infections with PA, characterized by 3+ mucoid and 3+ smooth forms. Lung specimen from a COPD dog that died of PA infection was provided by Dr. Miranda Vieson at the University of Illinois at Urbana-Champaign (UIUC) Veterinary Diagnostic Laboratory, as previously reported[1].

Mouse study approval. Mouse experiments were conducted in strict compliance with the guidelines outlined in the "Guide for the Care and Use of Laboratory Animals" by the National Institutes of Health. All mouse experiment procedures were approved by the Institutional Animal Care and Use Committee (IACUC) at the UIUC under Protocols 20120 and 23132.

Mouse models. All experiments were performed with 6-8-week-old age and sex-matched mice. C57BL/6 mice were procured from Charles River Laboratories. IL-17A KO mice (in C57BL/6 genetic background)[2] were originally provided to Dr. Nanjappa by Professor Yoichiro Iwakura (Tokyo University of Science, Tokyo, Japan). For the VOCs challenge, isoflurane-anesthetized mice (groups of 8) received intranasal instillation of specified concentrations of VOCs once daily for 1-3 weeks, using a mouse model of chronic inflammation[3-6]. Control mice were intranasally exposed to the same volume of vehicle (0.1% DMSO in PBS) for the same duration. The concentrations of VOCs intranasal instillation were based on the maximal levels detected in the headspace of bacterial culture[7, 8]: 2-AA (26 μg/mL), 1-undecene (317.5 ng/mL), 2-nonanone (22.4 ng/mL), and 1-dodecene (9.5 ng/mL). Mouse airways were evaluated for the degrees of GCM and mucin production using PAS, as well as IHC and/or IF analyses for MUC5AC, MUC5B, FOXA2, IL-17R, and IL-17A.

Sex as a biological variable. Our study examined male and female animals, and similar findings are reported for both sexes.

Depletion of macrophages and neutrophils. Systemic macrophage depletion was performed

according to the scheme presented in Fig. 7a. Clodronate liposome (CL) or control empty liposome (EL) (F70101C-N and F70101-N, respectively, FormuMax Scientific) was intraperitoneally injected at 10 μL/g mouse weight on Day -4, and subsequent injection of 5 μL/g intraperitoneally on Day 1 and 5. VOCs were administered intranasally once daily.

Neutrophils were depleted according to the scheme presented in Fig. 8a. Mice were intraperitoneally injected with rat anti-mouse Ly6G (Clone 1A8, BioXCell) on Days 0, 2, 4, and 6, prior to daily exposure to VOCs. Control mice received rat IgG2a isotype control (Clone 2A3, BioXCell). Confirmation of neutrophil depletion in the airways was conducted through flow cytometry. The mice were sacrificed on Day 8.

Flow cytometric analysis. Bronchoalveolar lavage fluid (BALF) and lungs were harvested from inoculated mice at 3 weeks post-exposure post-inoculation. Flow cytometric analyses were performed as described[9, 10]. Single-cell suspensions of BALF and lung homogenates were incubated with Fc Block (BD Biosciences) in FACS buffer (2% BSA and 0.05% NaN_3 in 1X PBS) for 10 min at 4°C followed by surface staining using fluorochrome-conjugated anti-mouse antibodies: anti-CD90.2 (Clone 30-H12), anti-CD11c (Clone N418), anti-F4/80 (Clone T45-2342), anti-NK1.1 (Clone PK136), anti-Ly6G (Clone 1A8), anti-MHC II (Clone M5/114.15.2), anti-CD11b (Clone M1/70) mAbs and Live/Dead Near IR Viability kit (Invitrogen). For staining iNOS and Arginase-1, cells were permeabilized with Cytofix/Cytoperm (BD Biosciences) followed by intracellular staining with fluorochrome-conjugated antibodies. For the determination of cytokine-producing cells, single-cell suspensions were re-stimulated with 50 ng/ml phorbol 12-myristate 13-acetate (PMA) and 500 ng/ml Ionomycin (Sigma-Aldrich) in the presence of GolgiStop (BD Biosciences) for 4 hours at 37°C before staining for surface markers. Intracellular cytokine staining

was performed using Cytofix/Cytoperm kit (BD Biosciences). All fluorochrome-conjugated antibodies were purchased from BD Biosciences, BioLegend, or Invitrogen. Stained cells were analyzed by Cytek Aurora (Cytek Biosciences), and data analysis by FlowJo software (v10.8.1; BD Biosciences). All statistical analyses were performed using Prism 9.4.1v (GraphPad) software. Values are Mean ± SD. *$p<0.05$; **$p<0.01$; ***$p<0.001$; ****$p<0.0001$ by two-tailed unpaired Student's t-test.

Airway cell cultures. Submerged and ALI cultures of the human bronchial epithelial 16HBE14o- cells were grown as previously published[5]. cultured in minimum essential medium (MEM) supplemented with 10% fetal bovine serum and 1% penicillin-streptomycin, maintained at 5% CO2 and 37°C. Cells at passage 3 were trypsinized, seeded onto 100 mm dishes, and cultured until reaching 70% confluency. For ALI cultures, 16HBE14o- cells at passage 2 were trypsinized and seeded onto Costar Transwell inserts with a pore size of 0.4 µm (Corning™3460, Corning) at a density of 5×10^5 cells in MEM. Cultures were maintained for 5 days with media added to both apical and basolateral chambers. On Day 6, media were removed, and complete PneumoCult-ALI media (05001, Stemcell) supplemented with PneumoCult-ALI supplement (05003), heparin (07980), and hydrocortisone (07925) were added to the basolateral chamber only to induce epithelial differentiation. Airway cells were exposed to VOCs for 24-48 hours and subjected to western blot or fluorescence microscopy analyses.

Periodic Acid-Schiff (PAS) and goblet cell score determination, immunofluorescence (IF), and immunohistochemistry (IHC) staining. Various staining, image acquisition, and quantification of mouse, human, and canine lung sections were performed as previously published[3, 5, 6]. For staining

of mouse lungs, after euthanasia, a cannula was inserted into the trachea, and the lung was instilled with 10% neutral-buffered formalin at a constant pressure (25 cm H_2O). After tracheal ligation, the inflated lung was immersed in 10% neutral-buffered formalin for 24 hours before paraffin embedding. Paraffin-embedded lungs were sectioned to 5 µm thickness and stained with PAS reagent (395B, Sigma-Aldrich). The degree of PAS-stained mucin expression was quantified using a 4-tier grading system described by Grundström et al [11]: Grade 0: No goblet cell observed per bronchiole. Grade 1: Up to 5 goblet cells per bronchiole. Grade 2: 5-20 goblet cells per bronchiole; Grade 3 More than 20 goblet cells per bronchiole. For IHC, lung sections were stained with primary antibodies against specific proteins and visualized using the VECTASTAIN ABC Kit (PK-4000). For IF, tissue sections were probed with anti-mouse secondary antibodies conjugated to Alexa Fluor®568 or anti-rabbit secondary antibodies conjugated to Alexa Fluor®488. Nuclei were counterstained with DAPI. PAS and IHC images were captured using an Olympus DP70 light microscope. IF images were obtained using a Nikon A1R confocal microscope and quantified using the NIS-Elements AR program.

Whole-cell, cytosolic, and nuclear protein preparations. Protein extraction and western blotting were performed as previously published [1, 3, 5, 12, 13]. Whole-cell extracts were prepared from airway epithelial cells utilizing the M-PER™ Mammalian Protein Extraction Reagent (78501, Thermo Fisher Scientific). Briefly, airway cells were suspended in the M-PER extraction kit, followed by incubation on ice, sonication for 10 seconds, and vortexing every 15 minutes for 1 hour. The lysate was centrifuged at 14,200 rpm for 20 minutes, and the resulting supernatant was collected as the whole cell extract. For the fractionation of cytosolic and nuclear proteins, the Nuclear & Cytoplasmic Extraction Kit (786-182, G-Biosciences) was employed. Briefly, airway cells were

resuspended in SubCell buffer I and lysis reagent, followed by a 10-minute incubation on ice. After centrifugation at 14,200 rpm for 10 minutes, the supernatant constituted the cytoplasmic fraction. The resulting pellets were then resuspended in nuclear lysis buffer, subjected to a 1-hour incubation on ice, and sonicated for 10 seconds every 15 minutes. The mixture was again centrifuged at 14,200 rpm for 20 minutes, and the supernatant was collected as the nuclear fraction. The protein concentration in each preparation was determined using the Pierce BCA Protein Assay Kit (#23227, Thermo Scientific).

Western blot analyses. Agarose-acrylamide hybrid gels were used to resolve high molecular weight MUC5AC and MUC5B mucins[1, 5, 12]. Regular 10-12% SDS-PAGE gels were used for all other proteins. To formulate agarose-acrylamide hybrid gels, a mixture of 1% agarose in TBE buffer was combined with 3-6% acrylamide, 150 mM Tris-HCl (pH 8.8), and 10% glycerol. The gel mixture was dissolved through repeated heating in a microwave. Before solidifying, SDS, ammonium persulfate, and TEMED were incorporated into the mixture. Gel electrophoresis was conducted using TBE buffer, and proteins were subsequently transferred onto a 0.45 μm PVDF membrane (88518, Thermo Fisher Scientific). For all other proteins separated on SDS-PAGE gels, the transfer was performed onto a 0.45 μm nitrocellulose membrane (88018, Thermo Fisher Scientific). After transfer, membranes were blocked with 5% skim milk dissolved in TBST and were then incubated overnight at 4°C with specific primary antibodies. The immune complexes were visualized with the ECL Western Blotting Detection System (34087, Thermo Fisher Scientific). In all Western blot analyses, primary antibodies were diluted as suggested by product datasheets.

Statistical analysis. Statistical analyses were conducted using GraphPad Prism 9.4.1V. and10, and quantitative data were presented as the mean ± standard error of the mean (s.e.m). In cases where equal variance was observed among samples, the significance between groups was assessed through a two-tailed Student's t-test. For samples exhibiting unequal variance, a two-tailed Welch's t-test was employed. In scenarios involving multiple comparisons, a one-way analysis of variance (ANOVA) followed by a post hoc Tukey's test was implemented. Significance levels were established as follows: $^{*}p \leq 0.05$, $^{**}p \leq 0.01$, $^{***}p \leq 0.001$, and $^{****}p \leq 0.0001$.

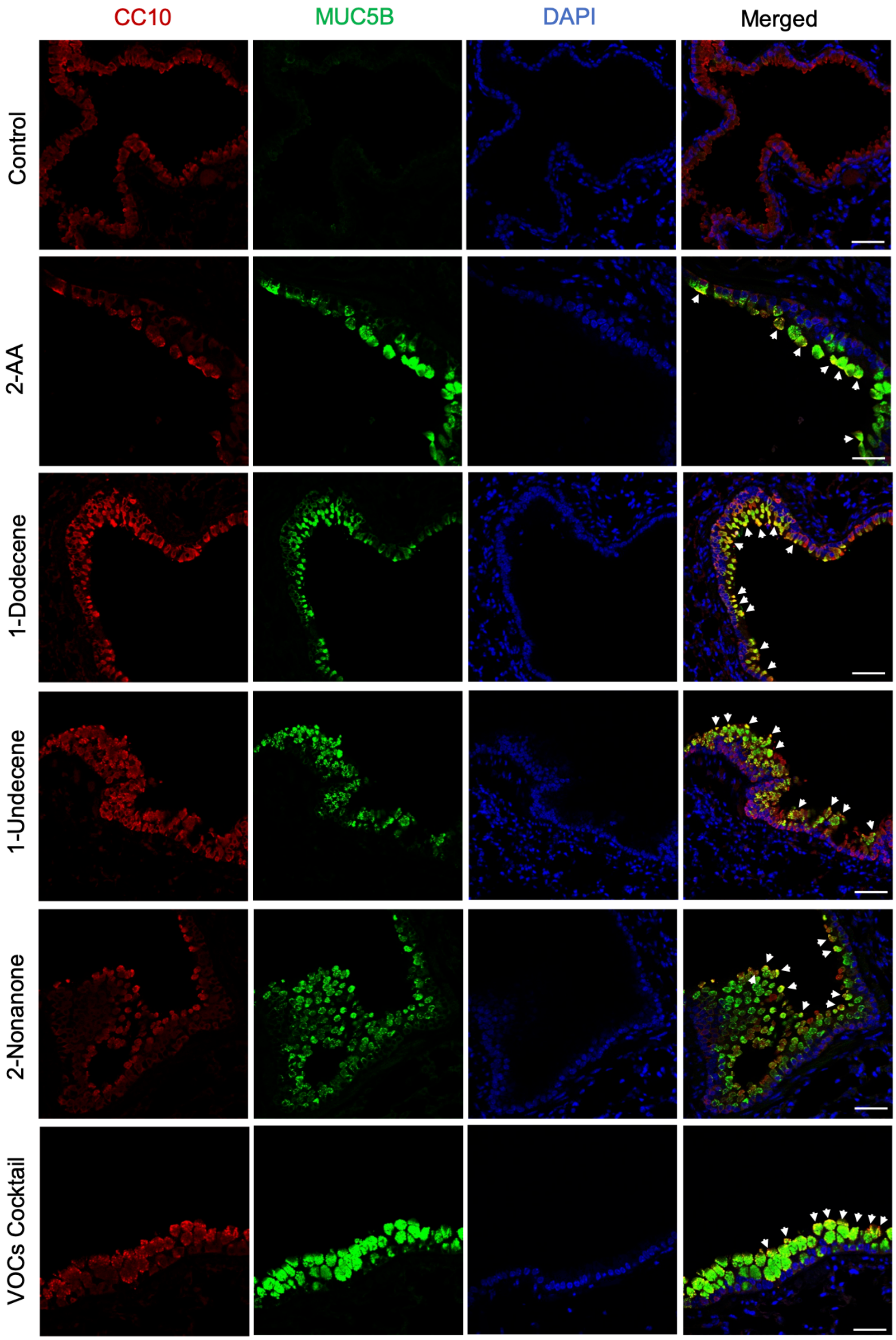

Supplementary Figure 1. PA-derived VOCs induce transdifferentiation of airway club cells into mucus-secreting goblet cells in murine lungs.

Lung sections of sex-matched C57BL/6 mice (groups of 8) exposed to vehicle control (0.1% DMSO in PBS), or PA VOCs were double-stained with specific antibodies against CC10 (club cell marker) and MUC5B (goblet cell marker). Representative images are shown. White arrows indicate double-positive cells. MUC5B was detected with an Alexa Fluor®488-conjugated secondary antibody (green). CC10 was detected using Alexa Fluor®647-conjugated secondary antibodies (red). Nuclei were counterstained with DAPI (blue). Scale bar = 50 µm. This figure set displays the individual fluorescence channels corresponding to the club cell–to–goblet cell axis shown in **Figure 2A**.

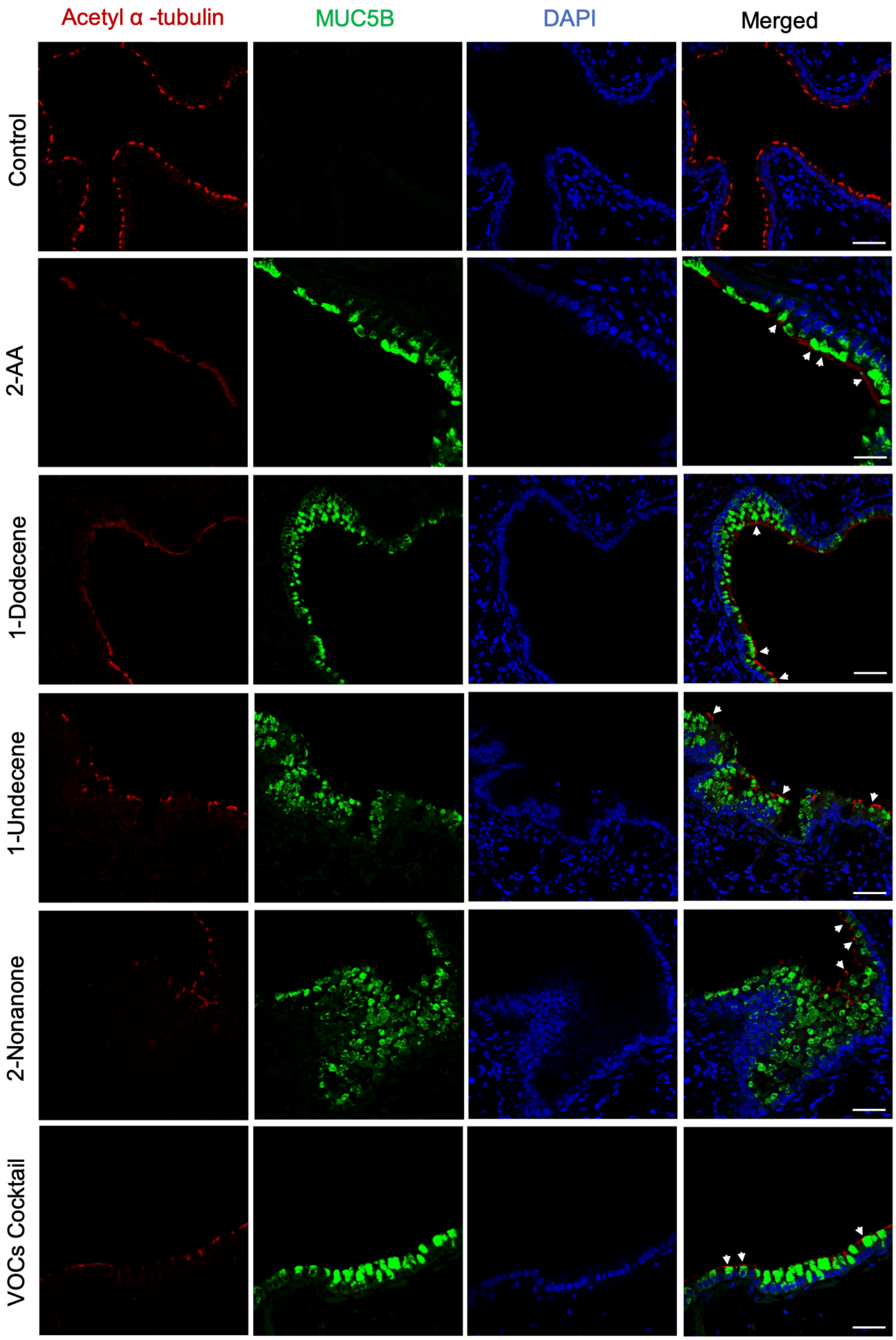

Supplementary Figure 2. PA-derived VOCs induce transdifferentiation of airway ciliated cells into mucus-secreting goblet cells in murine lungs.

Lung sections of sex-matched C57BL/6 mice (groups of 8) exposed to vehicle control (0.1% DMSO in PBS) or PA VOCs were double-stained with specific antibodies against CC10 (club cell marker) and MUC5B (goblet cell marker). Representative images are shown. White arrows indicate double-positive cells. MUC5B was detected with an Alexa Fluor®488-conjugated secondary antibody (green). CC10 was detected using Alexa Fluor®647-conjugated secondary antibodies (red). Nuclei were counterstained with DAPI (blue). Scale bar = 50 µm. This figure set displays the individual fluorescence channels corresponding to the ciliated cell–to–goblet cell axis shown in **Figure 2B**.

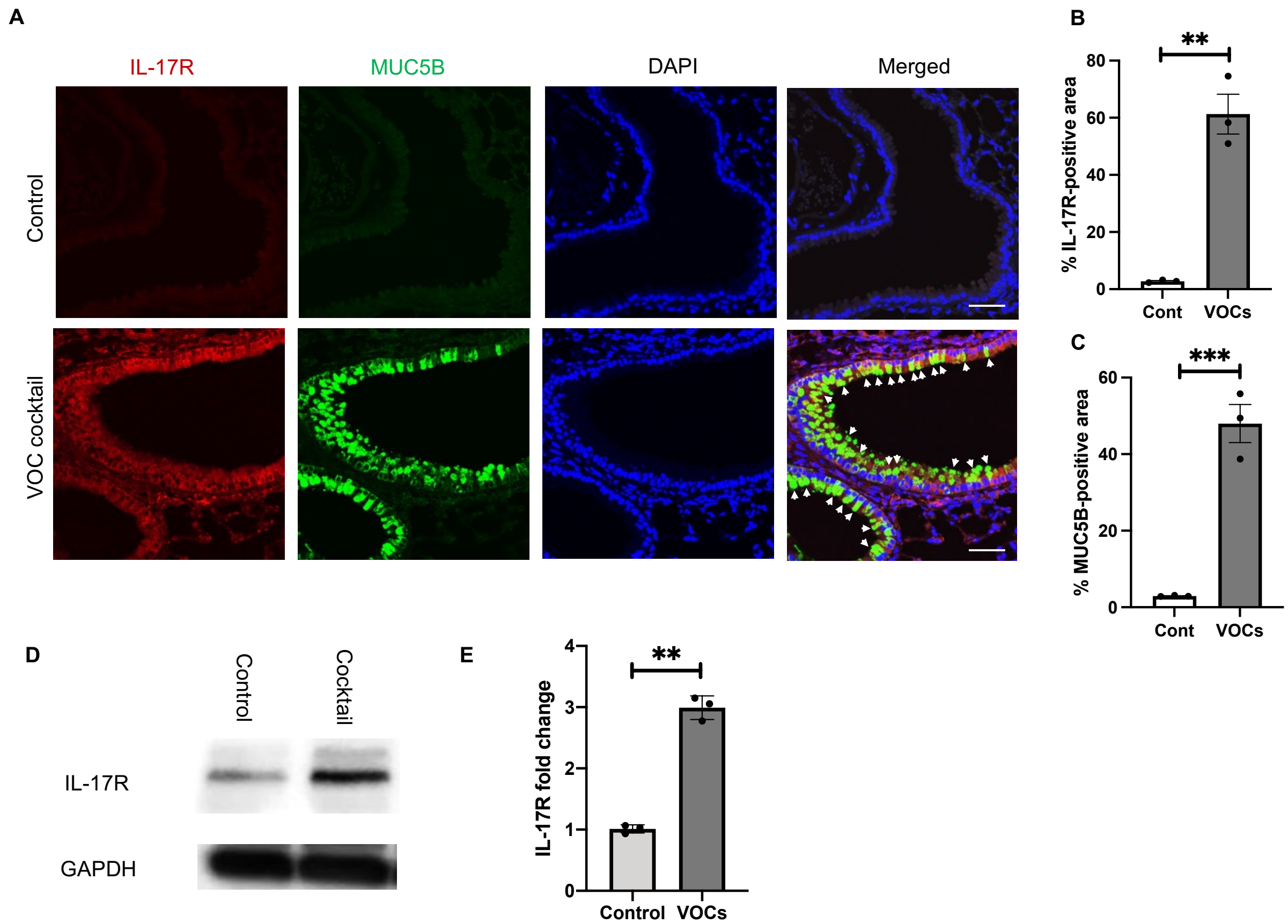

Supplementary Figure 3. PA-derived volatile organic compounds (VOCs) activate IL-17 receptor signaling in vivo and in vitro.

Sex-matched C57BL/6 mice (groups of 8) were intranasally exposed to vehicle control (0.1% DMSO in PBS; Cont) or VOCs cocktail once daily for 3 weeks. **A.** PA VOCs activate IL-17 receptor in murine airways with mucin overexpression. Mouse lungs challenged with PA VOCs or vehicle control were stained with primary antibody against IL-17R and MUC5B (goblet cell marker). Labeled airway sections were visualized by a secondary antibody conjugated to Alexa Fluor 594 (red, IL-17R) or Alexa Fluor 488 (cyan-green, MUC5AC), respectively. Nuclei were counterstained with DAPI (blue). Scale bar = 50 um. Representative sections are shown. White arrows indicate double-positive cells. **B–C.** Quantification of IL-17R and MUC5B immunofluorescence. IL-17R and MUC5B expression is presented as the percentage of IL-17A- and MUC5B-positive area and shown as mean ± s.e.m. $^{**}p < 0.01$, $^{***}p < 0.001$ versus control, as determined by a two-tailed Student's *t*-test (N=3). **D.** PA VOCs activate IL-17R in 16HBE14o- cells in vitro. Cells were exposed to VOCs for 24 hours, and IL-17R protein expression was assessed by immunoblotting. GAPDH served as a loading control. Experiments were repeated independently three times with similar results. **E.** Densitometry of IL-17R expression from western blots is shown as mean ± s.e.m. $^{**}p < 0.01$ versus PBS control, determined by two-tailed Student's *t*-test.

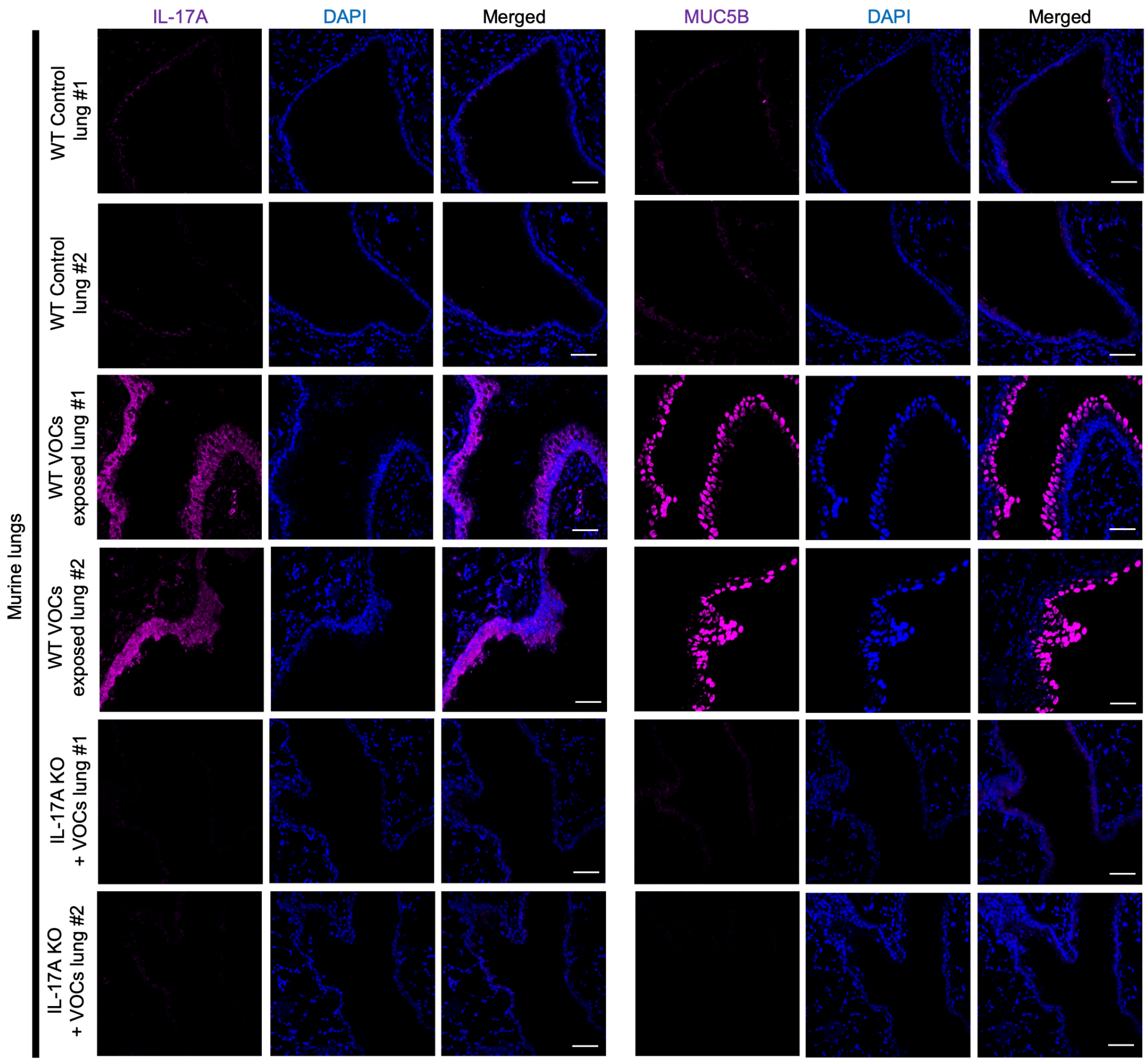

Supplementary Figure 4. IL-17A expression is upregulated in PA VOCs-exposed wild-type murine airways with mucus overproduction.

Additional replicates of lung sections from sex-matched wild-type (WT) and IL-17A-deficient mice (groups of 8) exposed to PA VOCs for one week were stained with primary antibodies against IL-17A and the goblet cell-specific marker MUC5B. Nuclei were counterstained with DAPI (blue). Immunolabeling was visualized using secondary antibodies conjugated to Alexa Fluor 647 (magenta, IL-17A and MUC5B). Replicate airway sections corresponding to **Figure 4** are shown. Scale bar = 50 µm.

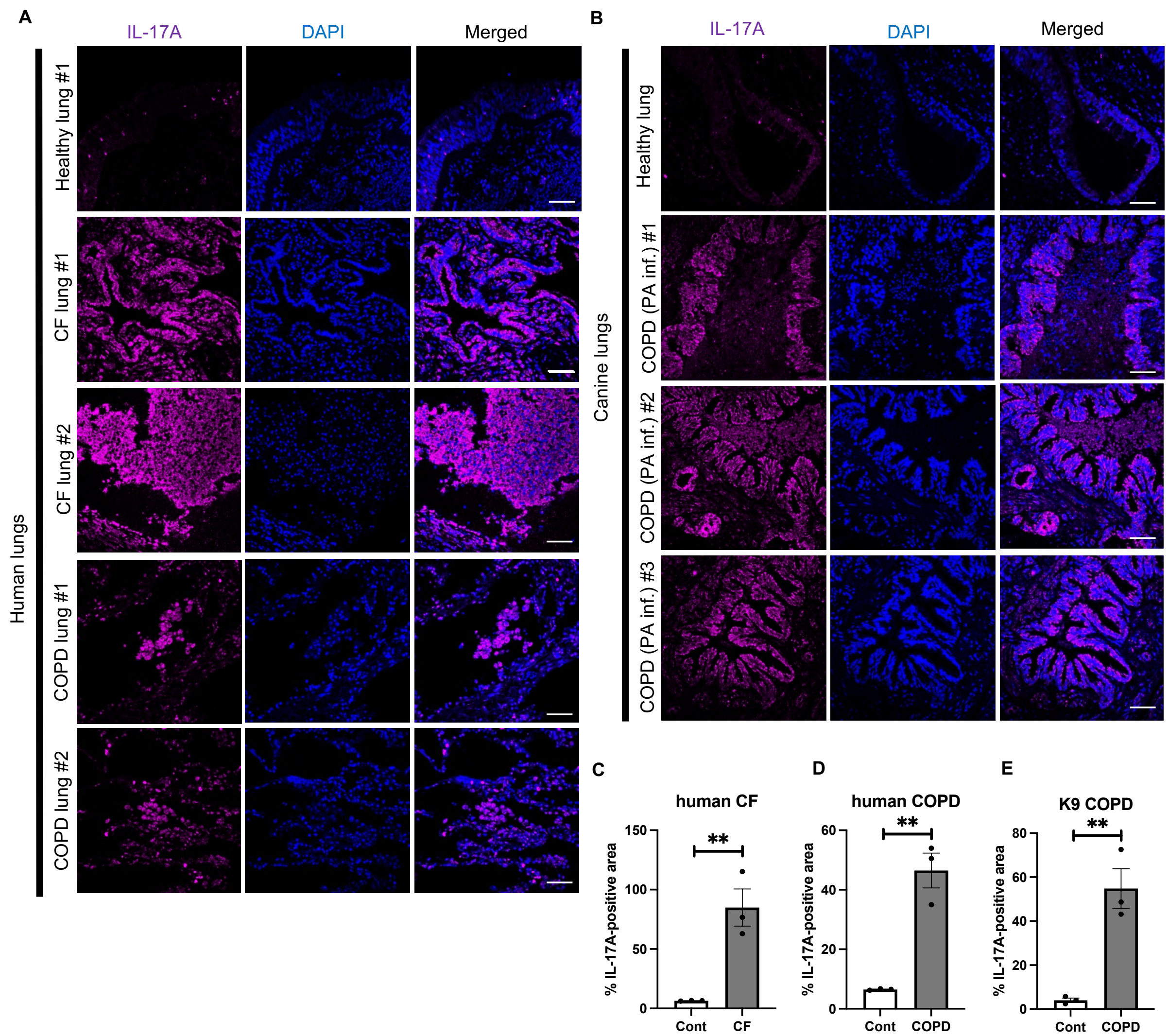

Supplementary Figure 5. IL-17A expression is upregulated in diseased human CF and COPD lungs and canine COPD airways that died of severe PA infection.

Lung sections from **(A)** humans and **(B)** dogs with PA airway infection were immune-stained with a primary antibody against IL-17A and detected with an Alexa Fluor 647-conjugated secondary antibody (magenta). Nuclei were counterstained with DAPI (blue). Representative images are shown. Scale bar = 50 µm. **(C–E)** Quantification of IL-17A immunofluorescence in human and canine airways. IL-17A expression is presented as the percentage of IL-17A-positive area and shown as mean ± s.e.m. **$p < 0.01$ versus control, as determined by a two-tailed Student's *t*-test. Abbreviations: CF, cystic fibrosis; COPD, chronic obstructive pulmonary disease.

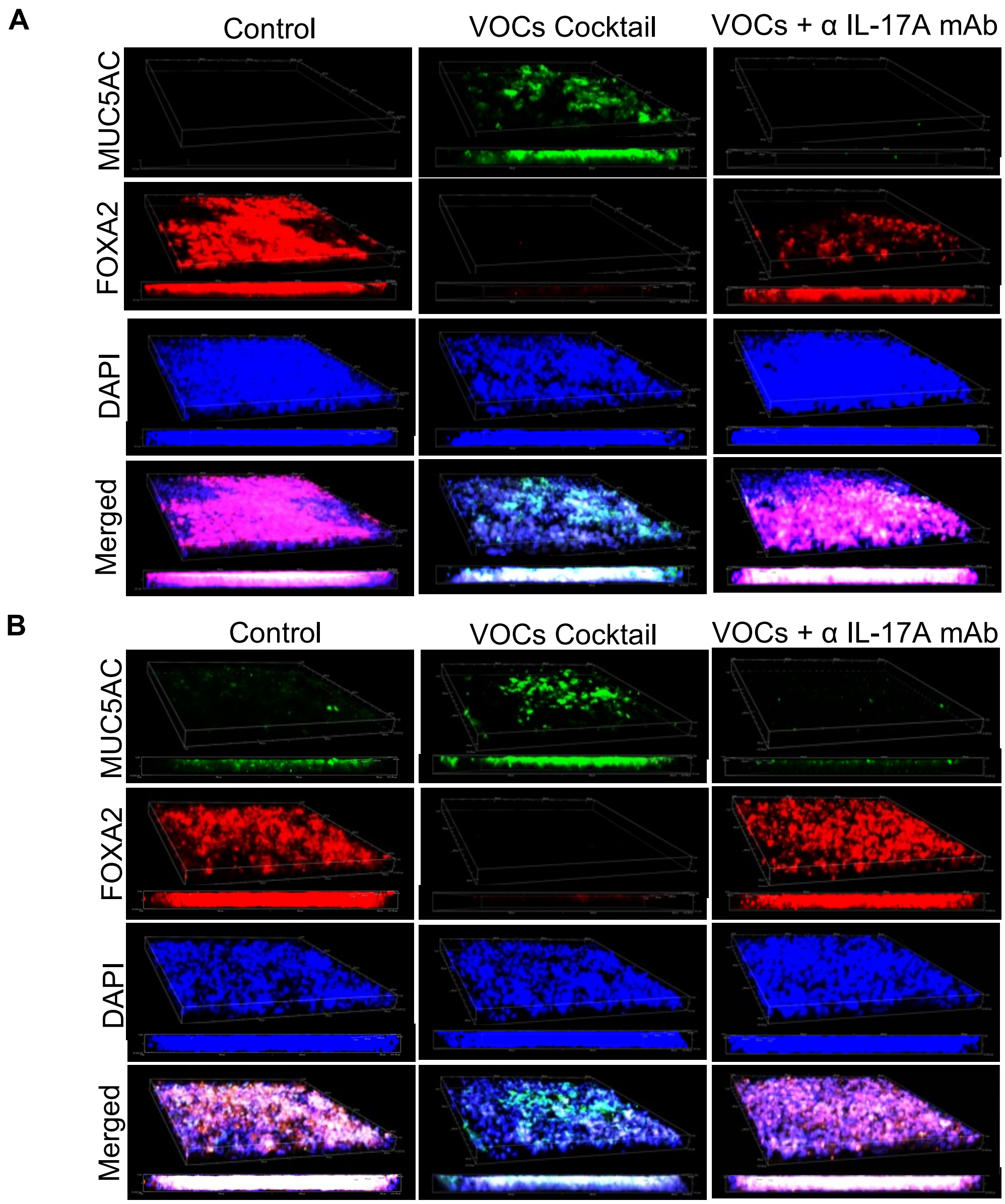

Supplementary Figure 6. Autocrine IL-17A induces mucus overproduction and FOXA2 degradation in ALI cultures of 16HBE14o⁻ cells exposed to PA VOCs.

Four-week-old, partially differentiated ALI cultures of human bronchial epithelial cells were potentiated to differentiate into mucin-secreting goblet cells by exposure to the PA VOCs cocktail for 48 hours in the presence or absence of the IL-17A neutralizing antibody (12047-M237, 1.0 µg/ml, one hour prior to VOC exposure). Cells were labeled with specific primary antibodies and visualized using secondary antibodies conjugated to Alexa Fluor 594 (red, FOXA2) or Alexa Fluor 488 (cyan-green, MUC5AC), followed by 3D and z-stack confocal microscopy. Experiments were independently repeated in triplicate. Replicate images corresponding to **Figure 5A** are shown in panels **A** and **B**.

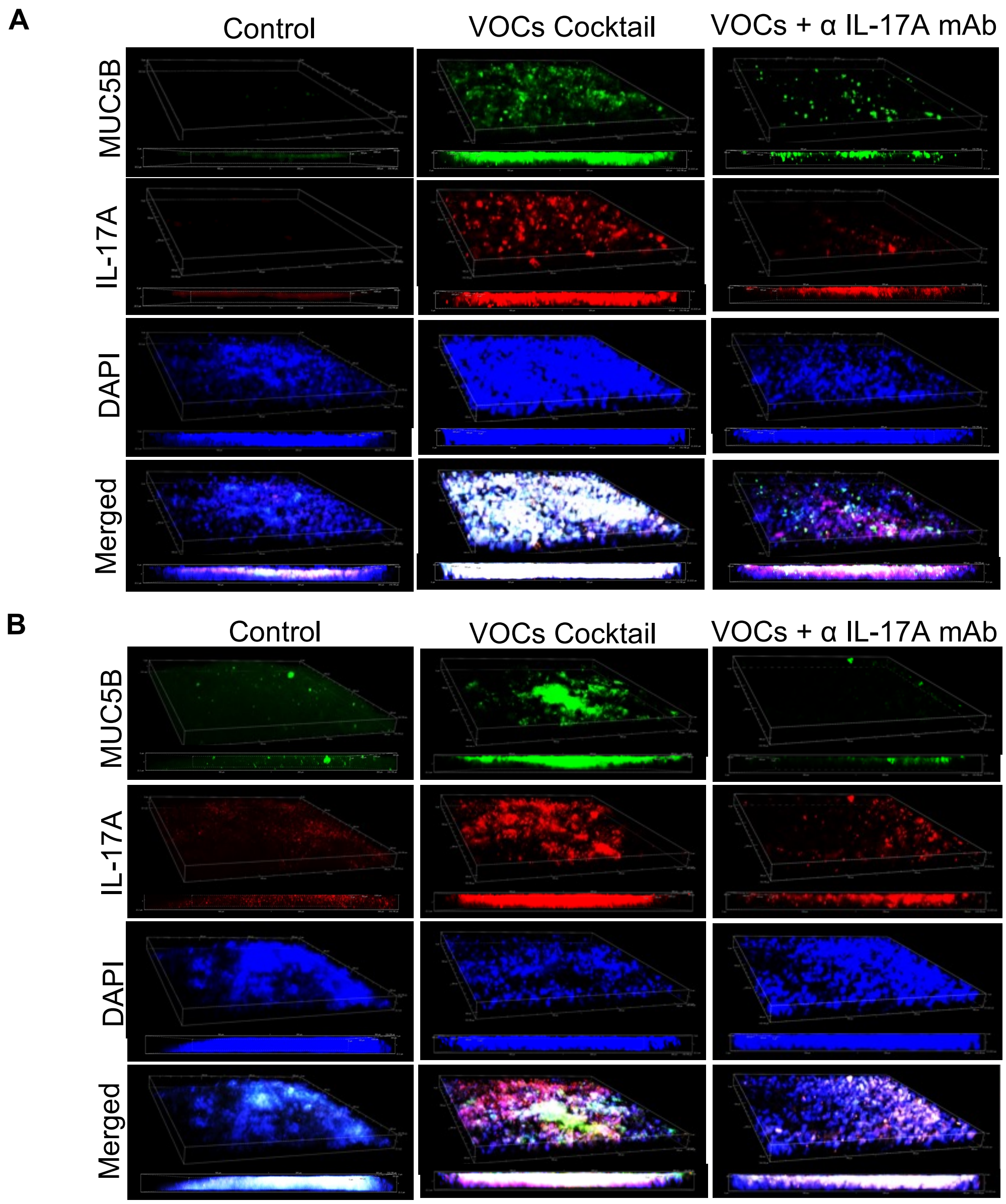

Supplementary Figure 7: Epithelium-derived IL-17A acts autocrinally to drive mucus hypersecretion in PA VOC-exposed ALI cultures of 16HBE14o⁻ cells.

Four-week-old, partially differentiated ALI cultures of human bronchial epithelial cells were potentiated to differentiate into mucin-secreting goblet cells by exposure to the PA VOCs cocktail for 48 hours in the presence or absence of the IL-17A neutralizing antibody (12047-M237, 1.0 μg/ml, one hour prior to VOC exposure). Cells were labeled with specific primary antibodies and visualized using secondary antibodies conjugated to Alexa Fluor 594 (red, IL-17A) or Alexa Fluor 488 (cyan-green, MUC5B), followed by 3D and z-stack confocal microscopy. Experiments were independently repeated in triplicate. Replicate images corresponding to **Figure 5B** are shown in panels **A** and **B**.

References

1. Choi W, Yang AX, Waltenburg MA, Choe S, Steiner M, Radwan A *et al.* FOXA2 depletion leads to mucus hypersecretion in canine airways with respiratory diseases. *Cell Microbiol* 2019; **21**(1)**:** e12957.

2. Cypowyj S, Picard C, Marodi L, Casanova JL, Puel A. Immunity to infection in IL-17-deficient mice and humans. *Eur J Immunol* 2012; **42**(9)**:** 2246-2254.

3. Hao Y, Kuang Z, Walling BE, Bhatia S, Sivaguru M, Chen Y *et al.* Pseudomonas aeruginosa pyocyanin causes airway goblet cell hyperplasia and metaplasia and mucus hypersecretion by inactivating the transcriptional factor FoxA2. *Cell Microbiol* 2012; **14**(3)**:** 401-415.

4. Caldwell CC, Chen Y, Goetzmann HS, Hao Y, Borchers MT, Hassett DJ *et al.* Pseudomonas aeruginosa exotoxin pyocyanin causes cystic fibrosis airway pathogenesis. *Am J Pathol* 2009; **175**(6)**:** 2473-2488.

5. Choi W, Choe S, Lin J, Borchers MT, Kosmider B, Vassallo R *et al.* Exendin-4 restores airway mucus homeostasis through the GLP1R-PKA-PPARgamma-FOXA2-phosphatase signaling. *Mucosal Immunol* 2020; **13**(4)**:** 637-651.

6. Jeffries JL, Jia J, Choi W, Choe S, Miao J, Xu Y *et al.* Pseudomonas aeruginosa pyocyanin modulates mucin glycosylation with sialyl-Lewis(x) to increase binding to airway epithelial cells. *Mucosal Immunol* 2016; **9**(4)**:** 1039-1050.

7. Filipiak W, Sponring A, Baur MM, Filipiak A, Ager C, Wiesenhofer H *et al.* Molecular analysis of volatile metabolites released specifically by Staphylococcus aureus and Pseudomonas aeruginosa. *BMC Microbiol* 2012; **12:** 113.

8. Cox CD, Parker J. Use of 2-aminoacetophenone production in identification of Pseudomonas aeruginosa. *Journal of clinical microbiology* 1979; **9**(4)**:** 479-484.

9. Sharma J, Mudalagiriyappa S, Abdelaal HFM, Kelly TC, Choi W, Ponnuraj N *et al.* E3 ubiquitin ligase CBLB regulates innate immune responses and bacterial dissemination during nontuberculous mycobacteria infection. *J Leukoc Biol* 2024; **115**(6)**:** 1118-1130.

10. Mudalagiriyappa S, Sharma J, Vieson MD, Nanjappa SG. GM-CSF(+) Tc17 cells are required to bolster vaccine immunity against lethal fungal pneumonia without causing overt pathology. *Cell Rep* 2022; **41**(4)**:** 111543.

11. Grundstrom J, Saarne T, Kemi C, Gregory JA, Waden K, Pils MC *et al.* Development of a mouse model for chronic cat allergen-induced asthma. *Int Arch Allergy Immunol* 2014; **165**(3)**:** 195-205.

12. Choi W, Yang AX, Sieve A, Kuo SH, Mudalagiriyappa S, Vieson M *et al.* Pulmonary Mycosis Drives Forkhead Box Protein A2 Degradation and Mucus Hypersecretion through Activation of the Spleen Tyrosine Kinase-Epidermal Growth Factor Receptor-AKT/Extracellular Signal-Regulated Kinase 1/2 Signaling. *Am J Pathol* 2021; **191**(1)**:** 108-130.

13. Hao Y, Kuang Z, Xu Y, Walling BE, Lau GW. Pyocyanin-induced mucin production is associated with redox modification of FOXA2. *Respir Res* 2013; **14**(1)**:** 82.
